# Identification of osmotic stress resistance mediated by *MdKAI2* in apple

**DOI:** 10.3389/fpls.2024.1467034

**Published:** 2024-12-05

**Authors:** Hongyang Guo, Aoxing Chen, Zhifeng Yang, Wenmao Yang, Xianpu Wang, Lili Xu

**Affiliations:** Xinjiang Production and Construction Corps, Shihezi University, Shihezi, China

**Keywords:** apple, karrikins, *MdKAI2*, gene function, osmotic stress resistance

## Abstract

KAR (Karrikin), a novel plant growth regulator, can be recognized specifically by plants and can activate resistance responses. MdKAI2 is the natural receptor of KARs in apple. Here, we report the identification of osmotic stress resistance in *MdKAI2* via the method of genetic transformation. The phenotypic traits, resistance indicators, and transcriptional and metabolic regulation of *MdKAI2* were identified. KAR1, a highly active form of KAR, markedly promoted the root growth of Gala cultivar tissue culture‒generated plants, possibly through increases in ABA and TZR contents and decreases in the GA3 content. *MdKAI2* was markedly upregulated by PEG stress and significantly promoted the growth of apple calli under nonstress conditions, whereas it was significantly inhibited under 20% PEG stress, as was cell death. MdKAI2 significantly increased the content of total flavonoids, the activity of reactive oxygen species (ROS)‒scavenging enzymes (SOD, POD and CAT), and the content of osmoregulatory substances (soluble protein, soluble sugars and proline). It also inhibited the MDA content and conductivity under osmotic stress. Differentially expressed genes (DEGs), including multiple transcription factors (TFs), such as *MYB*, *bHLH* and *AP2‒EREBP*, are significantly regulated by MdKAI2, and genes involved in the mitogen‒activated protein kinase (MAPK) signaling pathway play crucial roles in the regulation of plant resistance. In addition, pathways such as brassinosteroid (BR) biosynthesis and ABC transporters were downregulated, and the MAPK signaling pathway; plant‒pathogen interaction; cutin, suberin and wax biosynthesis; alpha‒linolenic acid metabolism; and phenylpropanoid biosynthesis were upregulated by MdKAI2. MdKAI2 significantly regulates the levels of lipids, amino acids, terpenoids, benzene, organic acids, carbohydrates, and alkaloids and is involved in the metabolic processes of amino acids, carbohydrates, nucleotides, lipids and secondary metabolites. Furthermore, MdKAI2 positively regulates fatty acids, esters, and terpenoids and negatively regulates metabolites of amino acids, amides and alcohols, and the MAPK signaling pathway may mediate this process. The study has provided a new direction for the industrial application of KAR1 in apples and resistance breeding based on the gene of *MdKAI2*.

## Introduction

KAR (Karrikin), a butylene hydroxylactone found in the smoke of burning plant materials, is considered a key germination trigger for many fire−prone species (Nelson et al., 2009). As novel plant growth regulators, KARs can be specifically recognized by plants to activate multiple resistance responses at relatively low concentrations, and this mechanism is common in the plant kingdom (Flematti et al., 2004; Sun et al., 2020). There are six natural molecular forms of KARs. KAR1 (3-methyl-2H-furo[2,3-c] pyran-2-one), a highly active molecular form (Nelson et al., 2009), has been the focus of some research advances that have focused on its biological function in model plants.

KAI2 (karrikin insensitive 2) is a natural α/β−hydrolase receptor of KAR1. When stimulated by exogenous KAR1, the ubiquitin ligases MAX2 and KAI2 localize to the plasma membrane were interacted (Khosla et al., 2020; Mizuno et al., 2021), which leads to the degradation of the specific inhibitor SMXL by ubiquitination to activate the transcription of downstream functional genes, such as genes involved in seed germination, branching, and root development (Wang et al., 2022). Although both the chemical structure and signal transduction pathways of KARs and SLs (strigolactones) are similar and share the same ubiquitin ligase MAX2, the mechanisms of seed germination, seedling development, and branching are independent and irreplaceable in some pathways (Waters et al., 2015).

KAR/KLs/D14L−ligand signaling may also shape the rice rhizomicrobiome composition by facilitating the biosynthesis of flavonoids (Nasir et al., 2020). Plant *KAI2* can be traced back to the bacterial *RsbQ* gene (Wang et al., 2022). Bacteria and fungi in the plant rhizosphere microdomain may have new receptors different from RsbQ and D14/KAI2, suggesting that butyrolactone may act as a signal transmitter between plants and rhizosphere microorganisms (Wang et al., 2022), which has positive and long−term implications for improving the root microdomain environment of fruit trees during the long growing period. KAI2 has been reported to positively regulate plant resistance. In *Arabidopsis thaliana* with *kai2* loss−of−function mutations, not only are the leaf stomata enlarged but also the permeability of the epidermis increases, which causes cell membrane damage under drought stress but also inhibits the synthesis of anthocyanins and reduces the sensitivity of stomatal closure and cotyledon opening to ABA hormones (Li et al., 2017). KARs improve the stress resistance of woody plants by influencing key genes involved in the ABA signaling pathway to increase the contents of endogenous sugars and amino acids and activate the ROS scavenging mechanism (Shah et al., 2020). In addition, KARs can also increase the synthesis and accumulation of flavonoid compounds by regulating SL biosynthesis and then recruit beneficial rhizosphere microorganisms to improve the microdomain environment, promoting plant growth and development, nutrient uptake and stress resistance (Nasir et al., 2020).

It has been confirmed in model plants that KARs can not only recruit beneficial microorganisms, improve the rhizosphere microenvironment, and increase soil water and fertilizer supply performance but also improve plant stress resistance. However, relevant studies of KARs, which are environmentally friendly plant growth regulators, are rare in perennial higher woody plants such as apple, and the gene functions and key downstream resistance signaling pathways involved are still unclear. In this study, via stable transgenic technology and multiomic analysis in apple plants, the gene function of *MdKAI2* was identified, and the transcriptional regulatory and metabolic pathways regulated by *MdKAI2* were revealed. The results provide reliable data for the application of KAR1.

## Results

### Effect of KAR1 on apple root development

**Figure 1:**
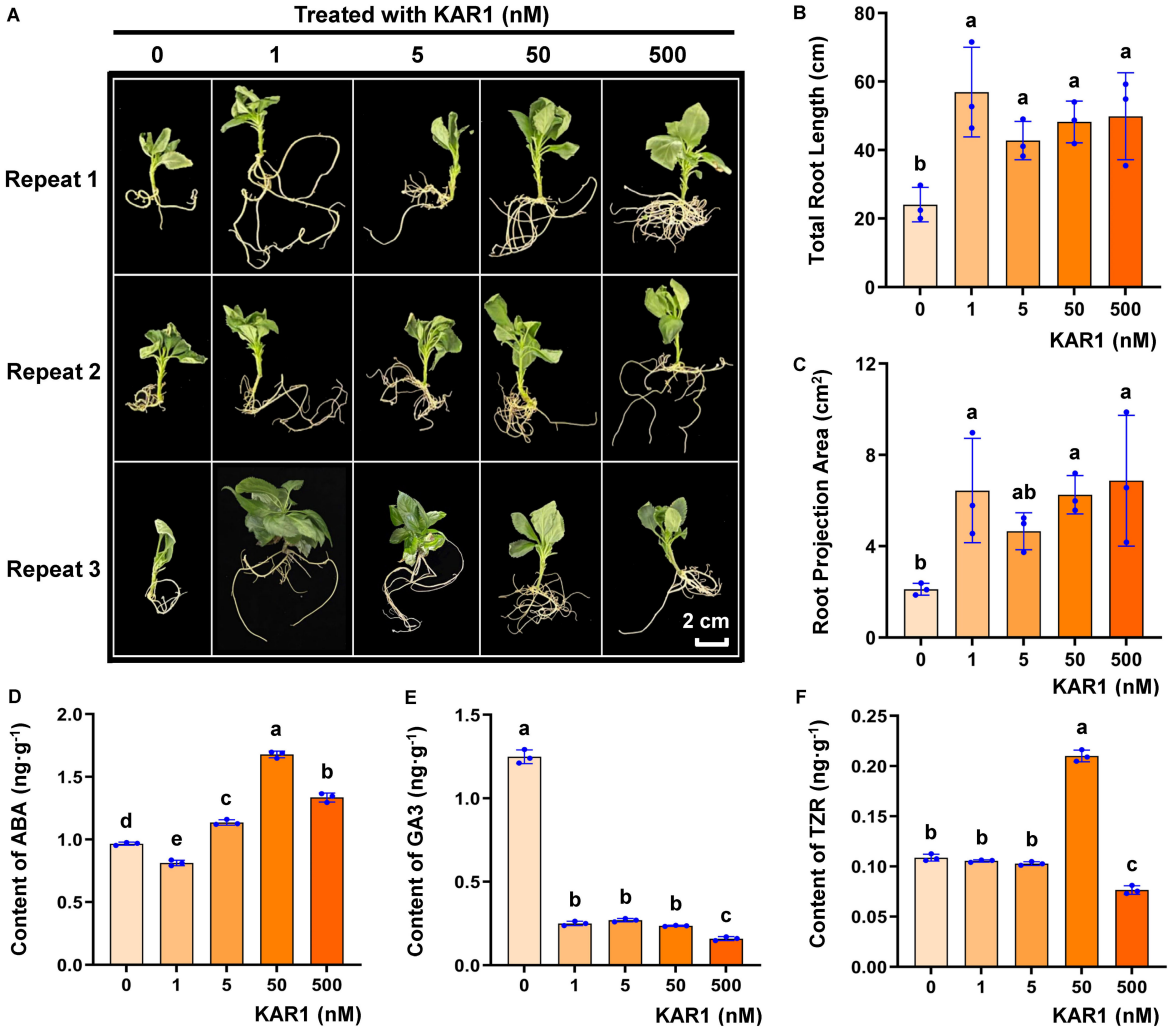
Effect of KAR1 treatment on the root development of Gala apple tissue culture-generated plants. In the treatment of different concentrations of KAR1 (0, 1 nM, 5 nM, 50 nM and 500 nM), IAA−induced rooting of Gala apple tissue culture−generated plants were conducted **(A)**. The concentration of IAA was 0.1 mg·L^-1^. Photographs were taken after photoperiod culture for 58 d (25°C, 16 h light and 8 h dark). The total root length **(B)** and root projection area were calculated **(C)**. The contents of the root hormones ABA **(D)**, GA3 **(E)**, and TZR **(F)** in apple seedlings subcultured with KAR1 for 4 days at concentrations of 0, 1 nM, 5 nM, 50 nM, and 500 nM were determined. Three independent experiments were conducted. One−way ANOVA and multiple comparisons via Fisher’s LSD test were performed, and different characters at the top of the columns indicate significant differences (p<0.05). The homoscedasticity and normal distribution of the data were confirmed by Levene’s test and the Shapiro−Wilk test, respectively. Scale bar = 2 cm.

Root development is a key factor for determining the growth and stress resistance of plants, especially fruit trees. To demonstrate the effect of KAR1 on apple root development, Gala apple tissue culture−generated plants were treated with different KAR1 concentrations (0, 1 nM, 5 nM, 50 nM and 500 nM) and rooted in ½ MS (Murashige and Skoog) media supplemented with 0.1 mg·L^-1^ IAA. KAR1 significantly promoted the development of taproots and lateral roots compared with those in the control group (**Figures 1A–C**).

Plant hormones play crucial roles in regulating root development. Furthermore, the contents of abscisic acid (ABA), gibberellic acid 3 (GA3), and trans−zeatin−riboside (TZR) plant hormones in the roots of apple seedlings subcultured for 4 days in MS media with KAR1 concentrations of 0, 1 nM, 5 nM, 50 nM, and 500 nM were determined. These results indicated that KAR1 significantly influences the endogenous hormone levels of apple roots (**Figures 1D–F**). The ABA content increased in the treatments with 5 nM, 50 nM, and 500 nM KAR1. The GA3 content decreased in the treatment range of 1 to 500 nM KAR1. The TZR content significantly increased in the 50 nM KAR1 treatment group but decreased in the 500 nM KAR1 treatment group. Treatment with 50 nM KAR1 had the most marked effect on roots, significantly increasing the ABA and TZR contents and reducing the GA3 content in apple roots, which indicated that KAR1−induced apple root development may be mediated by increasing ABA and TZR contents and decreasing the GA3 content.

### Phylogenetic analysis of the protein MdKAI2

Biological function is determined by molecular structure. Like that in *A. thaliana*, the natural receptor of KAR1 in apple, MdKAI2, is also conserved. Bioinformatics prediction revealed that MdKAI2 was a less stable weakly hydrophobic peripheral membrane protein (**Supplementary Figures S1A, C, D**) and had a greater content of alpha helices and random coils (**Supplementary Figure S1B**), which was related to its biological localization and function. In addition, a total of 9 sequence−conserved motifs (E value<0.05) were predicted among the 6 orthologous KAI2s from different Rosaceae plants (**Supplementary Figures S1E, F**). Several cis−acting elements related to plant hormone signals and stress resistance, including ABREs, EREs, and AREs, were also predicted in the promoter regions of the *MdKAI2* genes. In particular, multiple transcription factors (TFs) related to stress resistance, including MYB and MYC, are predicted to specifically bind to the promoter cis−elements of *MdKAI2* genes. Many basic cis−elements (TATA box and CAAT box) have also been predicted, which suggests that *MdKAI2* may be involved in the regulation of resistance and maintenance of high transcription levels when needed (**Supplementary Figure S3**).

**Figure 2:**
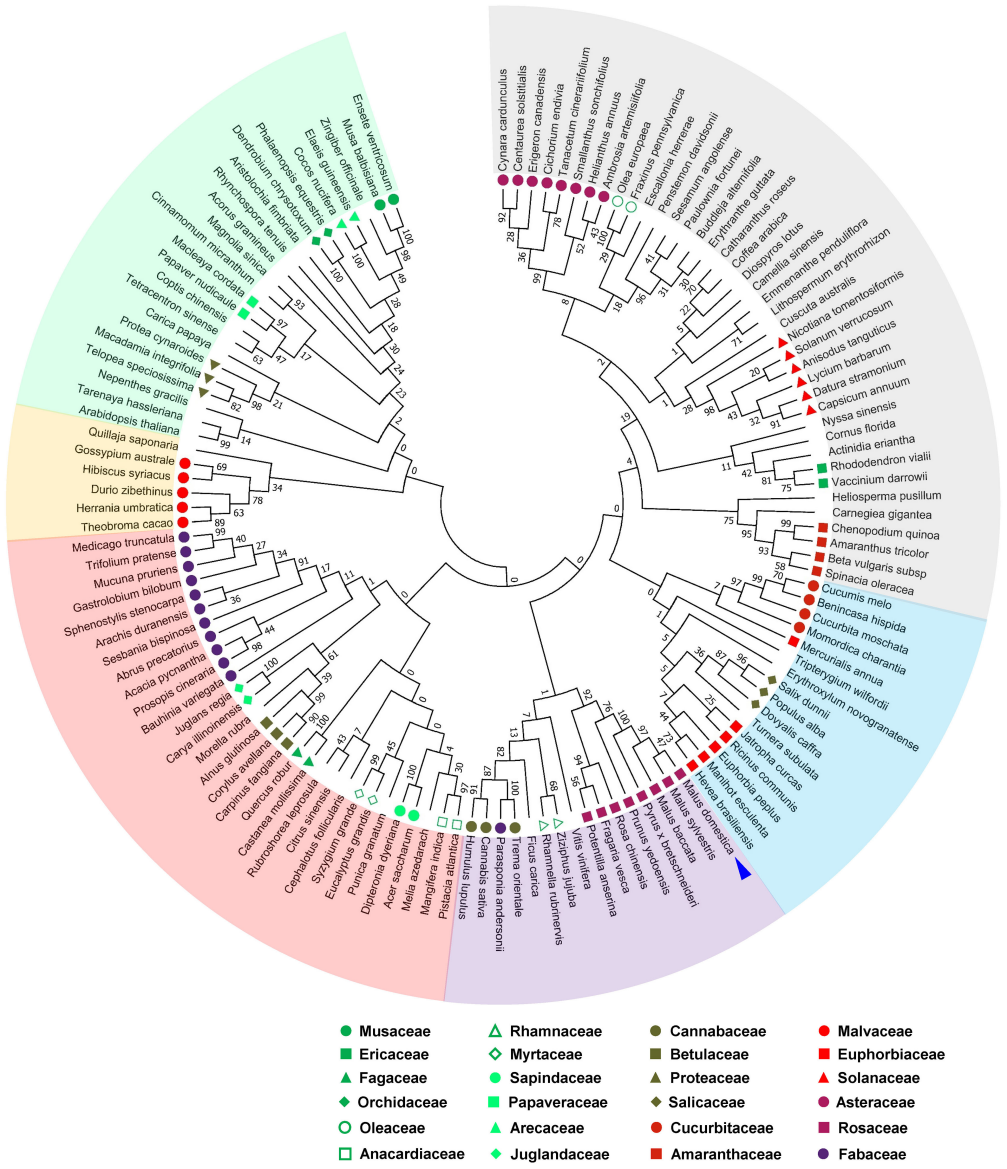
Evolutionary relationships among orthologous proteins of MdKAI2. Using the amino acid sequence of MdKAI2 as a template, orthologous proteins were aligned via the GenBank database, and 129 highly consistent orthologous genes were selected for analysis of their evolutionary relationships with those of *Malus domestica* and *A. thaliana*. The highlighted colors represent different groups. The species from which orthologous proteins originated are marked at the end of the branch. The symbol indicates the genus to which the species belongs (only the genus containing two or more orthologous proteins is marked). MdKAI2 is marked with a blue arrow. Evolutionary relationships were inferred via the neighbor−joining method, and the evolutionary distances were computed via the Poisson correction method.

The amino acid sequences of *A. thaliana* AtKAI2 and 129 KAI2s that are homologous to *Malus domestica* MdKAI2 were retrieved from the GenBank database and used to construct the phylogenetic tree. According to the phylogenetic relationships shown (**Figure 2**), the 131 KAI2 proteins can be divided into 6 groups and belong to 66 families. The genetic distance among members of the same genus is relatively short, and the genetic relationship is relatively close. Eight KAI2s belonging to the Vitis, Rhamnos, Moraceae, Cannabis, Fabaceae and Rosaceae taxa were clustered into one group. This extensive phylogenetic conservation among species implies the importance of the biological function of KAI2.

### Identification of the phenotype and physicochemical properties of transformed *MdKAI2* apple plants

**Figure 3:**
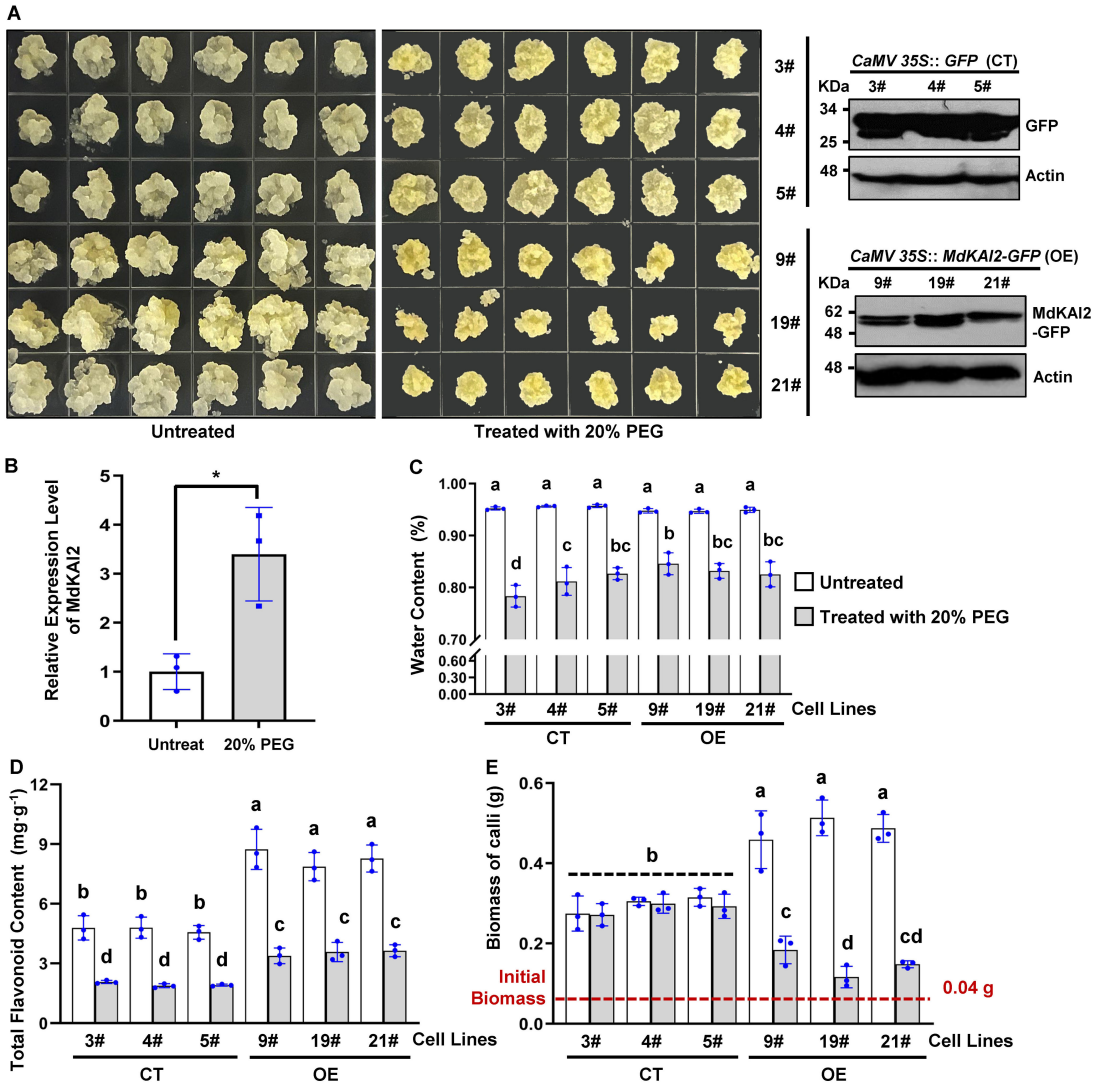
Phenotypic identification of the *MdKAI2* gene in apple calli. Three *MdKAI2*−*GFP*−overexpressing callus lines (OE lines, marked #9, #19 and #21) and three *GFP*−overexpressing callus lines (CT lines, marked #3, #4 and #5) were obtained via transfection of Orin apple calli and verified via western blotting **(A)**. Calli were inoculated in MS media vertically permeabilized with 20% PEG for 12 hours and subcultured in the dark at 24°C for 10 days to simulate osmotic stress. The control group without PEG stress was inoculated in MS media supplemented with the same volume of solvent, and the other operations were the same as those used for the stress group. Wild−type Orin apple calli treated with 20% PEG for 10 days were used for the detection of the *MdKAI2* expression level **(B)**. Phenotypes **(A)**, fresh weight biomass **(C)**, water content **(D)**, and flavonoid content **(E)** of calli with or without PEG stress were evaluated. One−way ANOVA and multiple comparisons via Fisher’s LSD test were performed. The different characters at the top of the columns indicate significant differences (p<0.05). Student’s t test with paired and two−tailed distributions for pairwise comparisons was conducted, and asterisks indicate significant differences (*p<0.05). Three independent experiments were conducted, and the individual points for each biological replicate are marked in each column. The homoscedasticity and normal distribution of the data were confirmed by Levene’s test and the Shapiro−Wilk test, respectively.

To investigate the biological function of resistance mediated by *MdKAI2*, Gala apple tissue culture−generated seedlings and Orin apple calli were used for genetic transformation. Two binary vectors, pBI121−*CaMV 35S*::*MdKAI2*−*GFP* and pBI121−*CaMV 35S*::*GFP*, were used for *Agrobacterium tumefaciens*−mediated apple transfection, and the positive lines were the OE and CT lines, respectively. Western blot (**Figure 3A**) and quantitative real−time PCR (qRT−PCR) (**Supplementary Figure S2**) assays were conducted to identify the positive transgenic callus lines.

To simulate osmotic stress, 20% polyethylene glycol (PEG) was used. In response to PEG stress, the endogenous expression level of *MdKAI2* significantly increased in wild−type apple calli (**Figure 3B**), which indicated that *MdKAI2* was markedly induced by external osmotic stress. The water content of the calli did not significantly differ with or without *MdKAI2* transfection but significantly decreased after treatment with 20% PEG (**Figure 3C**). Although the total flavonoid contents of calli were reduced in the presence of PEG stress, the total flavonoid contents in the *MdKAI2*−overexpressing lines were significantly greater than those in the CT lines under PEG stress or nonstress conditions (**Figure 3D**). Compared with that of the CT lines, the biomass of the *MdKAI2*−positive calli significantly increased without PEG treatment but significantly decreased under PEG stress (**Figure 3E**). These results were also reflected in the phenotypic traits (**Figure 3A**). Notably, some white cell death was observed on the surface of the CT lines under PEG stress, whereas white cell death was not observed in the OE lines. Our results indicated that MdKAI2 promoted the growth of apple calli under normal environmental conditions but inhibited their growth under stress conditions and increased the total flavonoid content to increase survival under relatively extreme stress.

**Figure 4:**
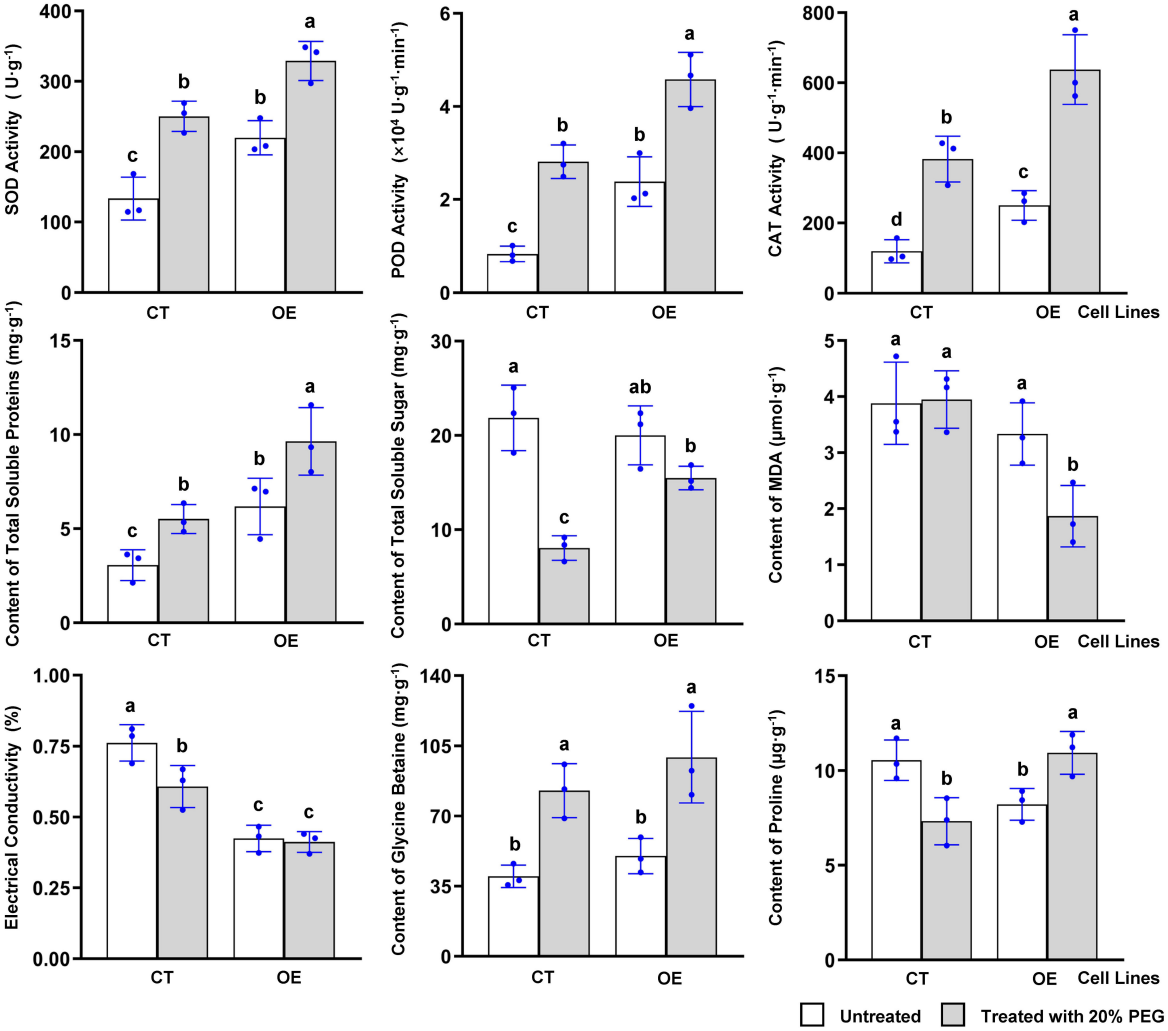
Effects of overexpressing *MdKAI2* on the main resistance indicators in apple calli. The following physicochemical indicators were detected: SOD, POD and CAT enzyme activity; total soluble protein, total soluble sugar, MDA, glycine betaine and proline contents; and electrical conductivity. Samples were collected after the transformed calli were cultured for 10 days. One−way ANOVA and multiple comparisons via Fisher’s LSD test were performed. Three independent experiments were conducted, and the individual points for each biological replicate are marked in each column. The different characters at the top of the columns indicate significant differences (p<0.05). The homoscedasticity and normal distribution of the data were confirmed by Levene’s test and the Shapiro−Wilk test, respectively.

To further investigate the physicochemical mechanisms by which MdKAI2 is involved in the regulation of plant resistance, several main indicators were investigated (**Figure 4**). The activity of reactive oxygen species (ROS)−scavenging enzymes, including SOD, POD, and CAT, increased after treatment with 20% PEG and further significantly increased in the *MdAKI2* transgenic callus lines, which indicated that the ROS balance mechanism was significantly promoted by MdKAI2 in apple cells. The level of malondialdehyde (MDA) and electrical conductivity are crucial indicators for evaluating the degree of oxidative damage in cells. The MDA content did not significantly differ among the CT lines and OE lines without PEG stress, whereas it significantly decreased in the OE lines under PEG stress. Although the electrical conductivity significantly decreased under PEG stress in the CT lines, the electrical conductivity markedly decreased in the OE lines.

Osmoregulatory substances are also markedly regulated by MdKAI2. Under PEG stress, the total soluble protein content significantly increased. Moreover, the contents of these genes were further elevated in the OE lines, and the contents in the OE lines reached the highest level. Under normal conditions, no significant differences in total soluble sugar content were detected between the CT and OE lines. The total soluble sugar contents of the CT lines significantly decreased in response to PEG, whereas there were no significant differences in the total soluble sugar contents of the OE lines. Moreover, under PEG stress, the total soluble sugar content in the OE lines was significantly greater than that in the CT lines. The proline content significantly decreased under PEG stress in the CT lines. In contrast, it significantly increased in the OE lines under PEG stress and reached an approximately equivalent level to that of the CT lines without PEG treatment. Although the content of glycine betaine significantly increased under PEG stress, no significant difference was detected between the CT and OE lines with or without PEG stress. These results demonstrated that MdKAI2 could promote the osmotic stress resistance of apple plants by increasing their ability to scavenge reactive oxygen species and facilitating the accumulation of osmoregulatory substances.

### Transcriptional regulation affected by MdKAI2 in apple calli

To analyze the transcriptional regulatory mechanism, Orin apple calli transformed with *MdKAI2*−*GFP* were subjected to RNA sequencing (RNA−Seq), and the CT group was used as the control. Three independent biological replicates were conducted. The average percentages of clean reads in the CT and OE groups were 93.85% and 93.74%, respectively, which met the analysis requirements.

**Figure 5:**
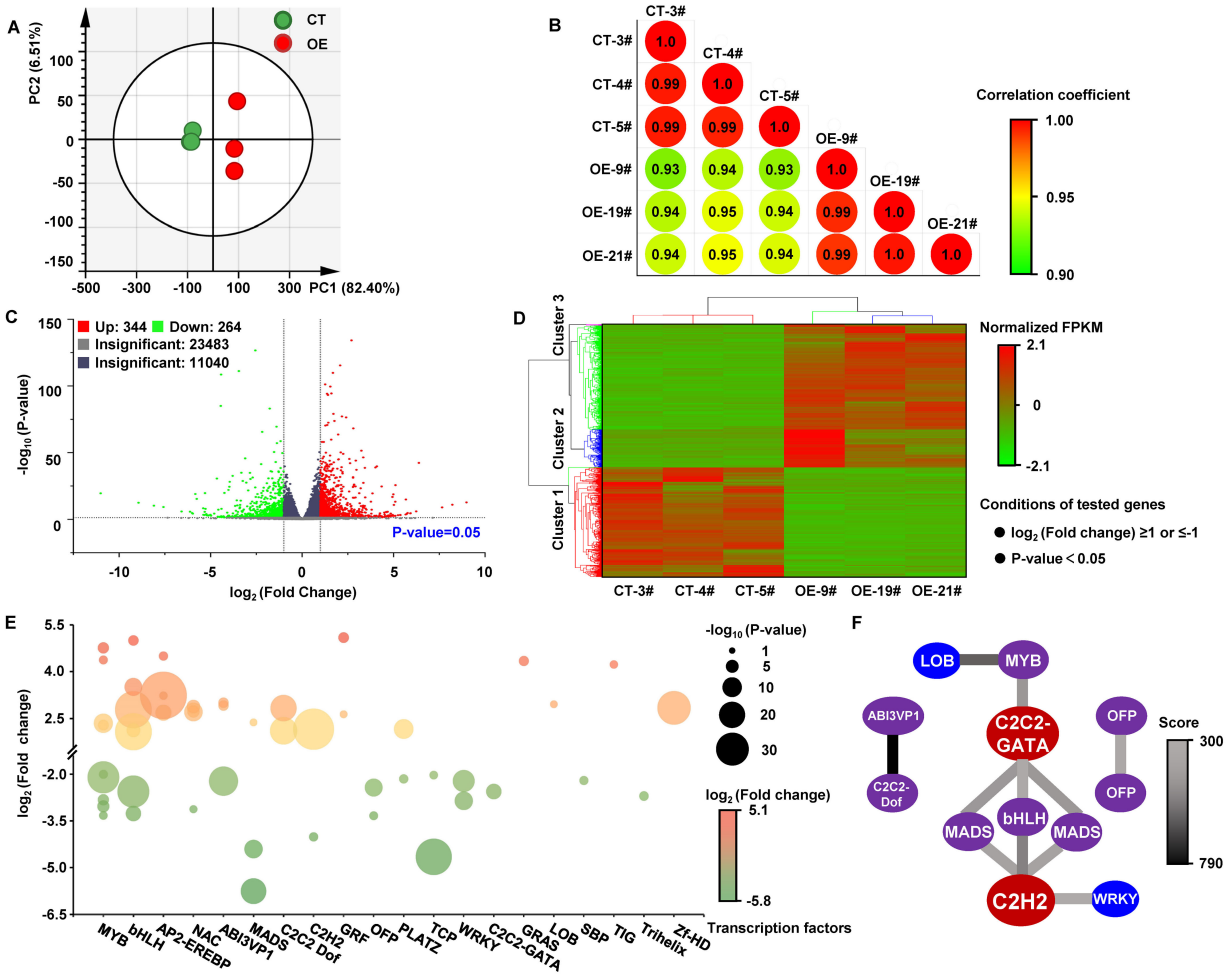
RNA−Seq analysis and DEGs in *MdKAI2*−overexpressing apple calli. Total RNA from *MdKAI2*−*GFP* (OE)− and *GFP* (CT)−overexpressing calli was used for RNA−Seq. Three independent experiments were conducted. **(A)** PCA of the transcriptomes was performed on the basis of the expression level. **(B)** Correlation analysis of the transcriptomes was performed on the basis of the expression level, and the correlation between samples was calculated via the Spearman correlation coefficient. **(C)** Volcano plot of the number of DEGs. **(D)** Cluster heatmap of DEGs. Differential genes with a log_2_ (fold change) ≥1 or ≤-1 and a P value<0.05 were used for cluster analysis. The fold change represents the gene FPKM value ratio of the OE and CT groups. The fragments per kilobase of transcript per million mapped reads (FPKM) values were standardized via the z score method to construct a cluster heatmap. **(E)** Analysis of differentially expressed annotated TFs according to the TF protein domain via the PlantTFDB (Plant Transcription Factor Database). Red and green represent up− and downregulated TFs, respectively, after overexpressing *MdKAI2*. The color depth represents the difference multiple, and the circle size represents the size of the P value. **(F)** Interaction network prediction of TFs. Using the BGI online program, the TFs were aligned to the STRING database, and the interactions between genes were obtained according to their homology with known proteins. The color of the line segment indicates the interaction score. The darker the color is, the more reliable the interaction.

PCA (principal component analysis) revealed that the intergroup difference was significant (**Figure 5A**), and the intragroup correlations were greater than the intergroup correlations (**Figure 5B**), which implied that the significant difference in the transcriptional level was due mainly to *MdKAI2* transformation. A total of 34132 transcripts were detected, and 344 transcripts and 264 transcripts were up− and downregulated, respectively (log_2_ (fold change)≥1 or ≤-1, P value<0.05; **Figure 5C**). A total of 608 differentially expressed genes (DEGs) were divided into three clusters on the basis of their expression abundance (**Figure 5D**). Cluster 1 represented the downregulated DEGs, whereas cluster 2 and cluster 3 represented the upregulated DEGs. The DEGs in Cluster 2 were expressed slightly more highly in the CT lines than those in Cluster 3 were.

In addition, a total of 51 TFs belonging to 20 types of TFs were screened according to a log_2_ (fold change) ≥ 2 or ≤ -2 and a P value < 0.05. These TFs were significantly up− or downregulated by *MdKAI2* overexpression (**Figure 5E**). The numbers of genes belonging to the *MYB*, *bHLH*, *AP2*−*EREBP*, and *NAC* families were relatively large (**Supplementary Table S3**). Moreover, the expression levels of representative TFs, including *MdWRKY*, *MdbHLH*, *MdAP2*−*EREBP*, *MdNAC* and *MdMYB*, which are closely associated with plant resistance, were verified via qRT−PCR (**Supplementary Figure S4**). These results were essentially in accordance with the RNA−Seq results and demonstrated that MdKAI2 significantly regulates the expression of multiple crucial TFs. Interaction network prediction of differentially expressed TFs was conducted (**Figure 5F**), and bHLH, MADS, MYB, WRKY, C2C2−GATA, C2H2 and LOB TFs exhibited strong interaction relationships.

**Figure 6:**
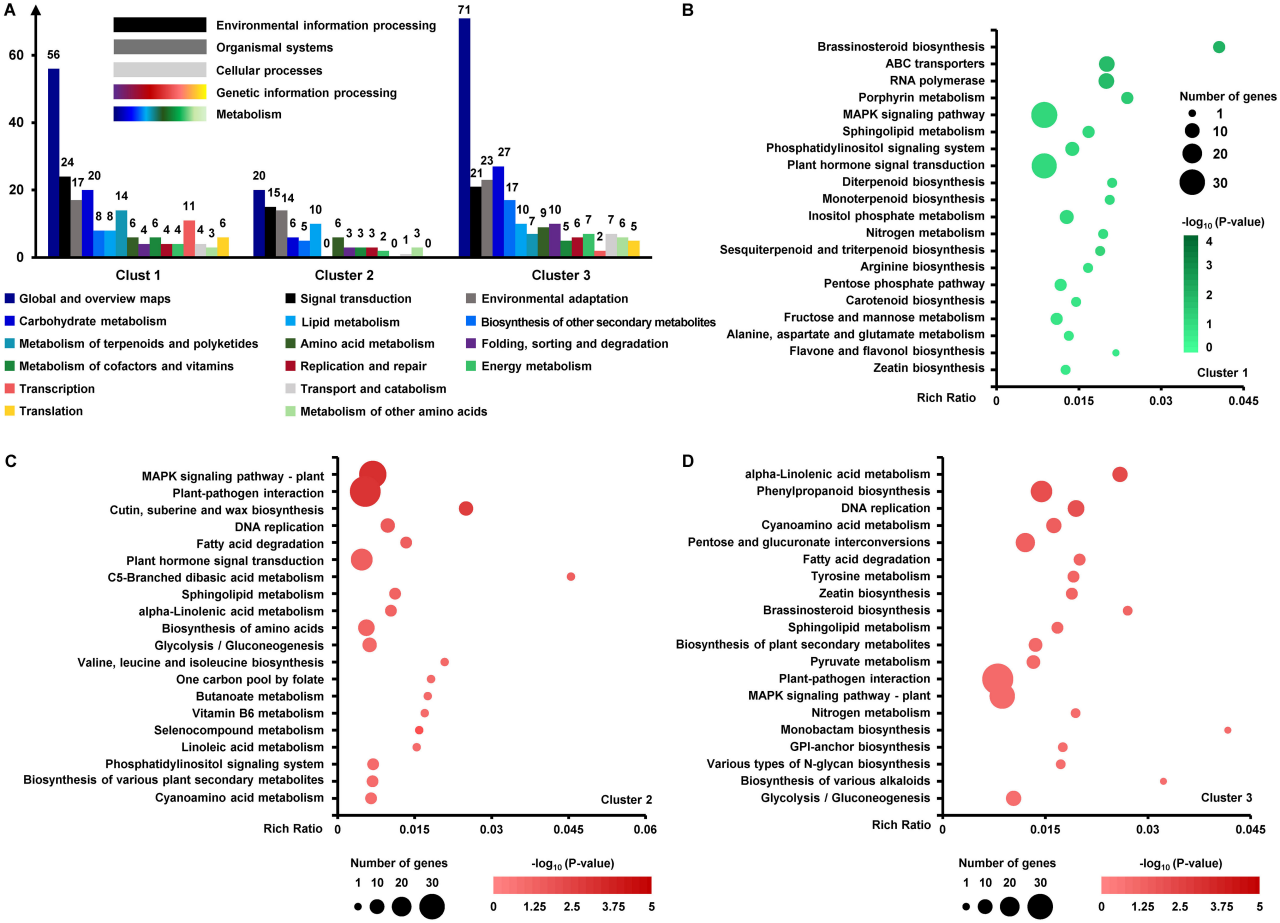
KEGG enrichment and classification analyses of DEGs. According to the results of the cluster analysis of the DEGs, KEGG classification analysis **(A)** was conducted on the up− and downregulated genes. The classification of gene clusters for which the sum of genes annotated to each classification was greater than ten was selected for statistical graphing. The color of the column indicates the different categories of the KEGG classifications, and the numbers on each column indicate the number of DEGs annotated to each classification in the gene cluster. KEGG enrichment analyses were conducted on the basis of one cluster of downregulated **(B)** and two clusters of upregulated **(C, D)** DEGs. The top 20 terms with low P values of enriched genes were selected for mapping. The rich ratio represents the ratio of the number of term candidate genes to the number of term genes. DEGs with log_2_ (fold change) values ≥1 or ≤-1 and a P value < 0.05 were utilized for analyses, and the fold change represents the ratio of the FPKM values of the OE and CT genes. The analyses above were conducted via OmicShare online programs.

According to the cluster analysis based on the expression levels of DEGs (**Figure 5C**), the two clusters of upregulated DEGs and one cluster of downregulated DEGs induced by *MdKAI2* overexpression were analyzed via Kyoto Encyclopedia of Genes and Genomes (KEGG) enrichment analysis and classification analysis. The DEGs were involved mainly in the pathways of the global and overview maps; environmental adaptation; signal transduction; and carbohydrate, lipid, amino acid terpenoid, polyketide and other secondary metabolism (**Figure 6A**).

The downregulated DEGs (cluster 1) were associated mainly with brassinosteroid (BR) biosynthesis, ABC transporter, RNA polymerase, porphyrin metabolism, and the MAPK signaling pathway. These genes are primarily responsible for regulating plant growth and development, promoting reproductive growth, modulating stomatal activity to achieve equilibrium water potential, enhancing light energy performance, inducing the synthesis and accumulation of stress−resistant substances, and strengthening the capacity to defend against both biotic and abiotic stresses.

The upregulated DEGs of cluster 2 were associated mainly with pathways such as the MAPK signaling pathway; plant−pathogen interaction; cutin, suberin and wax biosynthesis; DNA replication; fatty acid degradation; and plant hormone signal transduction (**Figure 6B**). These genes are involved mainly in regulating the plant signal response and transduction, reducing transpirational water loss, and enhancing pathogen defense ability and lipid metabolism. The upregulated DEGs of cluster 3 are related mainly to pathways including alpha−linolenic acid metabolism, phenylpropanoid biosynthesis, DNA replication, cyanoamino acid metabolism, and pentose and glucuronate interconversions (**Figure 6C**).

The processes related to these DEGs involve mainly lipid and amino acid metabolism and predominantly enhance the fluidity and stability of membrane lipids, govern the construction of cell walls and stress defense mechanisms, facilitate the synthesis and transduction of stress signals, and strengthen the defense against natural enemies and other multiple resistance mechanisms; these pathways may be involved in multiple signal transduction processes related to MAPK.

### Metabolite regulation by MdKAI2 in apple calli

**Figure 7:**
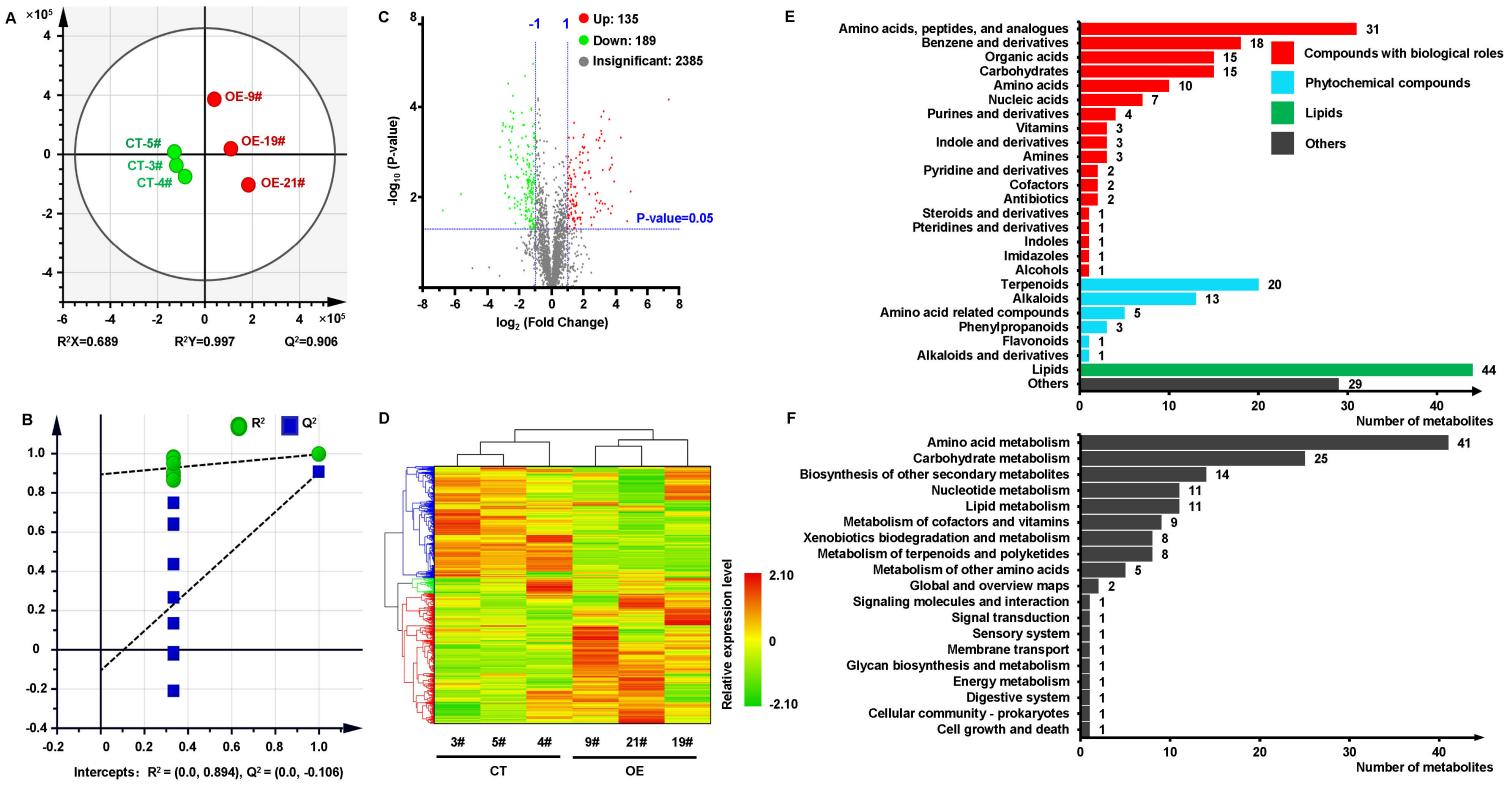
Metabolomic analysis of apple calli overexpressing *MdKAI2*. A total of 2709 metabolites were subjected to PLS−DA analysis **(A)**, and 200 permutation tests were performed to verify the R−squared value and predictive ability of the model **(B)**. The distributions of the up− and downregulated metabolites were analyzed **(C)**, and cluster analysis was performed on the basis of the relative abundance of the differentially expressed metabolites within the Pearson correlation coefficient (DEM), the metabolite abundance in each row was normalized via the z score method, and the average linkage method was used for clustering **(D)**. KEGG classification analysis **(E)** and pathway analysis **(F)** were conducted on all annotated metabolites via the OmicShare online program. Different colors represent different metabolite classification entries, and the numbers indicate the number of annotated metabolites in the final classification.

The plant metabolites are closely related to the plant defensive response. Orin apple calli transformed with *MdKAI2*−*GFP* were subjected to metabolome analysis, and the CT group was used as the control group. As shown by the PLS−DA (partial least squares discriminant analysis) results, metabolites with different abundances were more significantly different between the CT and OE groups, and the analysis model did not overfit the data (**Figures 7A, B**). A total of 2709 metabolites with different abundances were detected, and 135 metabolites and 189 metabolites were up− and downregulated, respectively (log_2_ (fold change) ≥1 or ≤ -1, P value < 0.05, **Figure 7C**). The expression levels of 2709 metabolites markedly differed in response to *MdKAI2* overexpression, and the metabolites could be divided into three groups on the basis of their differences in abundance (**Figure 7D**). Among the 324 differentially expressed metabolites (DEMs), 37 (**Supplementary Table S1**) were annotated in the KEGG database and were used for KEGG enrichment analysis (**Figures 7E, F**). The 37 annotated DEMs were mainly related to amino acids or peptides or their analogs, benzene and derivatives, organic acids, carbohydrates, terpenoids, alkaloids and lipids and are involved in lipid, nucleotide, secondary metabolite, carbohydrate, and amino acid metabolism processes, which were basically consistent with those of the KEGG analyses of RNA−Seq. The above results indicated that metabolites and metabolic processes, which are related primarily to amino acids, peptides and their derivatives, nucleic acids, ester compounds, and carbohydrates, were markedly affected by *MdKAI2* overexpression.

### Correlation analysis of metabolites and transcripts involved in the CDPK−MAPK signaling pathways

According to the results of the KEGG analyses of the DEGs (**Figure 6**), the MAPK signaling pathway may involve multiple resistance pathways induced by *MdKAI2* overexpression. The cross−talk between calcium−dependent protein kinases (CDPKs) and MAPK is important for regulating plant−specific responses to biotic and abiotic stresses (Ludwig et al., 2005). To further investigate whether some associations exist between CDPK−MAPK signaling pathways and these DEMs, *MdKAI2* and 41 differentially expressed MdMAPK signaling−related DEGs combined with 37 annotated DEMs were utilized for correlation analysis. In response to *MdKAI2* overexpression, the expression levels of two *MdCDPK*s were upregulated 4.8− and 8.8−fold, that of ten MAPK signaling−related DEGs was significantly downregulated, that of eight MAPK signaling−related DEGs was significantly upregulated, and the expression levels of representative DEGs related to MAPK signaling were verified via qRT−PCR (**Supplementary Figure S4**).

**Figure 8:**
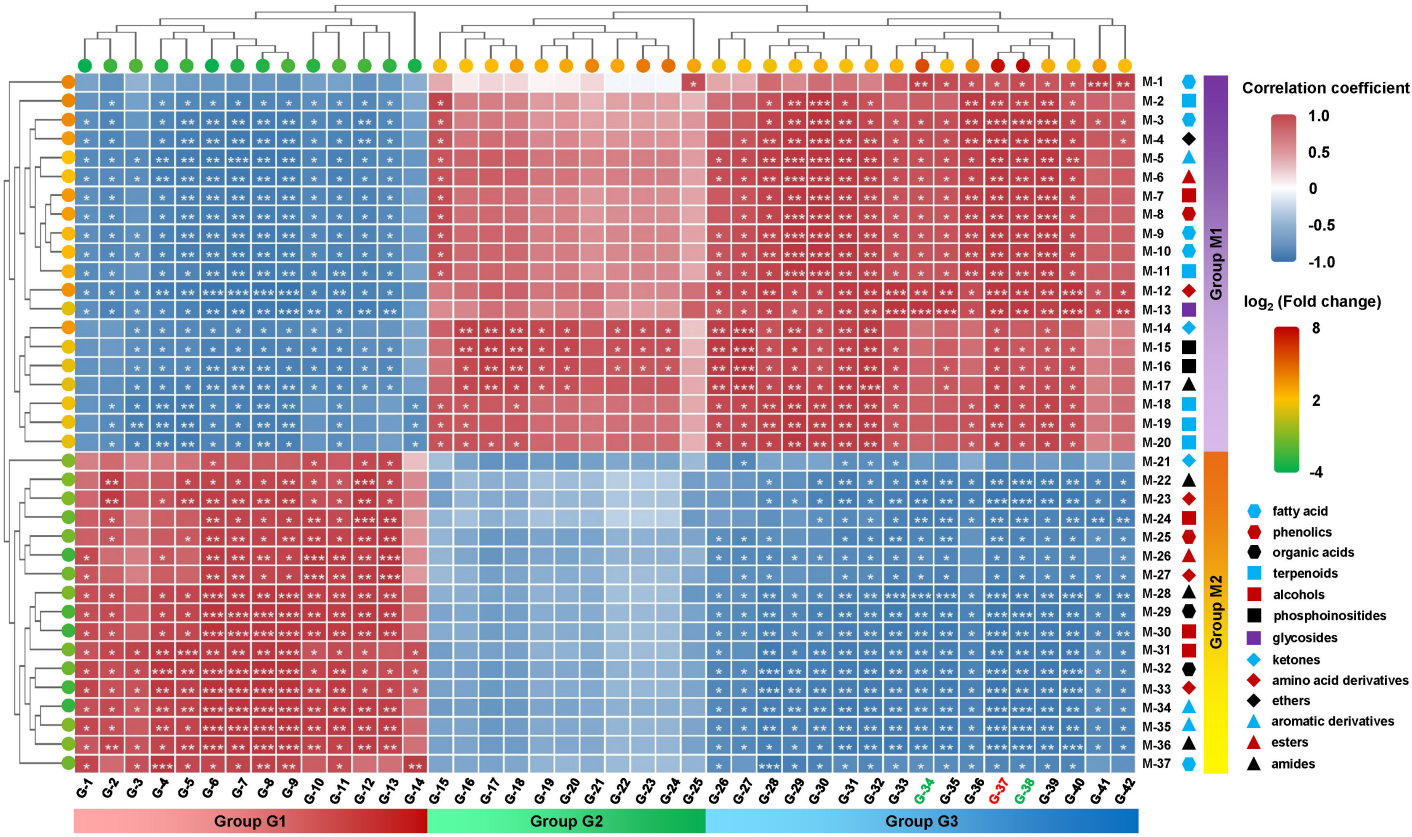
Correlation analysis of DEMs with *MdKAI2* and MAPK signaling pathway genes. Thirty−seven annotated DEMs (log_2_ (fold change) ≥ 1 or ≤ -1, P value <0.05, and VIP >1) were selected. A total of 42 DEGs (log_2_ (fold change) ≥ 2 or ≤ -2, P value <0.05), including genes related to the *MdKAI2* and MAPK signaling pathways, were also selected for correlation cluster analysis on the basis of their expression levels. The Pearson correlation coefficient was used to reflect the correlation. The color of the square color block indicates the correlation level, and the red and blue colors indicate positive and negative correlations, respectively. The asterisk indicates a correlation (*P< 0.05, **P < 0.01, ***P < 0.001). The circles on the left and above indicate the changes in the expression levels of genes and metabolites after the overexpression of *MdKAI2*. Red and green indicate up− and downregulation, respectively. The different colors and shapes of the icons below indicate the metabolite type. The code numbers on the right and below indicate the corresponding DEGs and DEMs, respectively.

**Figure 9:**
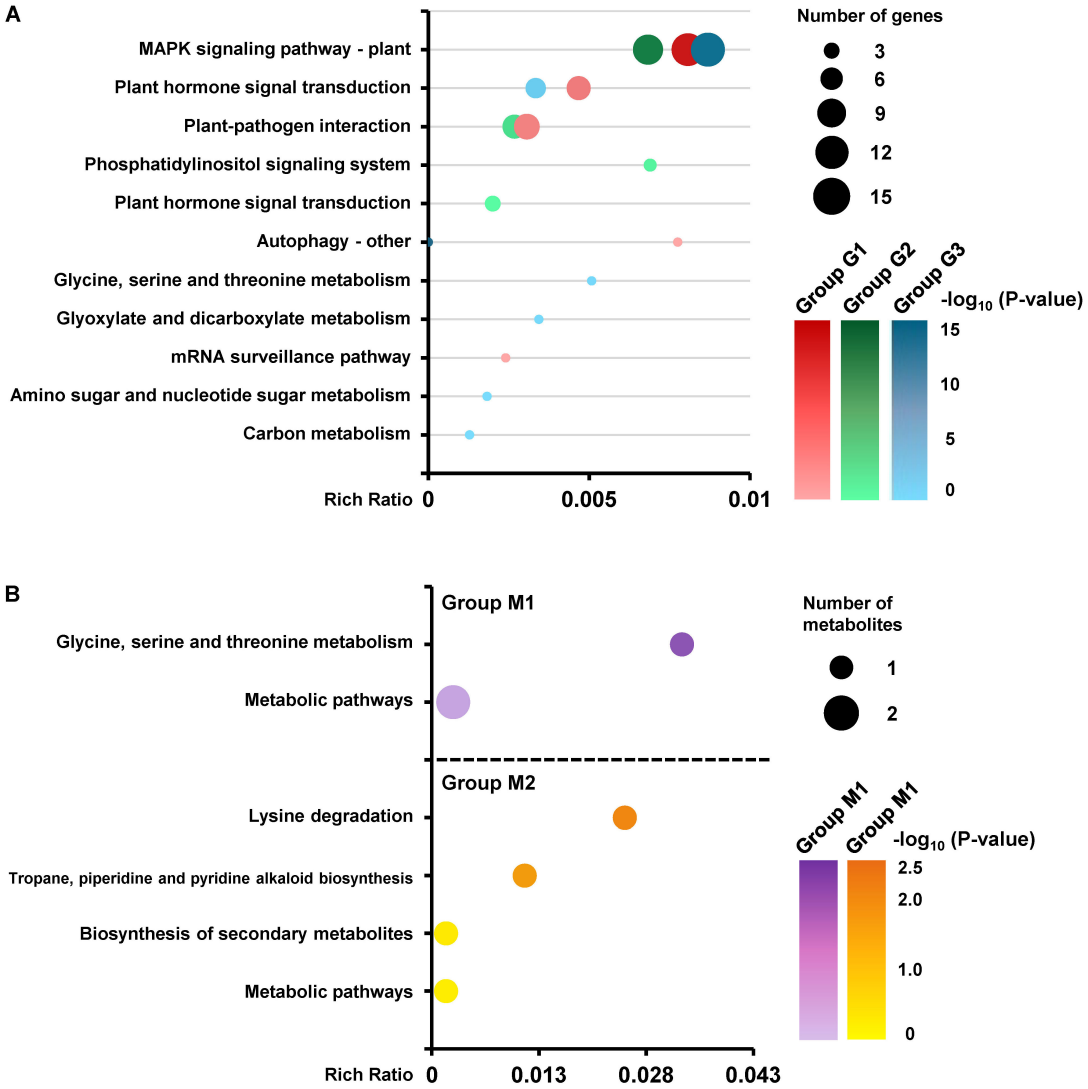
KEGG enrichment analyses of DEGs and DEMs. Two differentially expressed *MdCDPK*s, 39 differentially expressed MAPK signaling−related DEGs, and *MdKAI2*, whose expression levels were log_2_ (fold change) ≥ 2 or ≤ -2 and whose P value was <0.05, were used for KEGG enrichment analyses **(A)**. Thirty−seven annotated DEMs whose expression levels were log_2_ (fold change) ≥ 1 or ≤ -1, P value <0.05, and VIP >1 were used for KEGG enrichment analyses **(B)**. The order is sorted by P value from low to high. Different colors represent different groups of DEGs and DEMs, and the color shading indicates the P value. The sizes of the circles represent the number of DEGs and DEMs belonging to each corresponding pathway.

The results of the correlation analysis (**Figure 8**) revealed that 37 DEMs were significantly correlated with the MAPK signaling−related DEGs. These genes were involved in the MAPK signaling pathway, plant hormone signal transduction, and plant−pathogen interactions (**Figure 9A**), and the DEMs were involved mainly in multiple secondary metabolic processes, such as amino acid metabolism (**Figure 9B**).

Specifically, induced by *MdKAI2* overexpression, DEMs labeled as group M1 (mainly fatty acids, esters, and terpenoids) were significantly upregulated (**Figure 8**) and involved metabolic pathways such as glycine, serine, and threonine metabolism (**Figure 9B**), and other DEMs labeled as group M2 (mainly acid derivatives, amides and alcohols) were downregulated (**Figure 8**) and mainly participated in metabolic pathways such as lysine degradation; tropane, piperidine, and pyridine alkaloid biosynthesis; and the biosynthesis of secondary metabolites (**Figure 9B**). The DEGs associated with the MAPK signaling pathways can be classified into three categories (labeled as groups G1, G2, and G3).

The downregulated DEGs of group G1, which included multiple protein kinases, phosphatases, and TFs, including *MYC*, *WRKY*, and *bHLH*, were significantly positively correlated with those of group M2 DEMs and significantly negatively correlated with those of group M1 DEMs. These genes may positively regulate metabolites of amino acid derivatives, amides and alcohols by being involved in lysine degradation and the biosynthesis of tropane, piperidine, pyridine alkaloids and other secondary metabolites, while these processes are negatively regulated by *MdKAI2* overexpression (**Figures 8**, **9B**; **Supplementary Table S2**). The upregulated DEGs of group G3, which included multiple protein kinases, including *CDPK*, and TFs, including *MYC*, *bHLH* and *ERF*, were significantly positively correlated with those of group M1 DEMs and significantly negatively correlated with those of group M2 DEMs. These genes may positively regulate metabolites of fatty acids, esters, and terpenoids via glycine, serine and threonine metabolism, and these processes are positively regulated by *MdKAI2* overexpression (**Figures 8**, **9B**; **Supplementary Table S2**). In addition, the upregulated DEGs of group G2, including genes encoding calcium−binding proteins and protein kinases, may be involved in the positive regulation of some fatty acids, esters, and terpenoids and were not significantly correlated with DEMs, including amino acid derivatives, amides and alcohols (**Figure 8**; **Supplementary Table S2**).

## Discussion

KARs were initially identified for their ability to stimulate seed germination, and homologous plant analogs have not been found (Wang et al., 2022; Flematti et al., 2015). Studies have demonstrated that the KAR signaling pathway significantly influences seedling photomorphogenesis, leaf development, and tolerance to abiotic stress (Wang et al., 2022; Flematti et al., 2015; Nelson et al., 2010). KARs can significantly influence plant root development. Our study revealed that KARs significantly enhanced the root development of apple tissue culture−generated plants under nonstress conditions and extended the length of both the main and lateral roots (**Figure 1**), which is consistent with the results of Shah et al. for *Sapium sebiferum* (Shah et al., 2020). The root development of *S. sebiferum* was significantly improved by treatment with 1 nM KAR1 and 1 to 500 nM KAR1 in apple tissue culture−generated seedlings. These results were also consistent with those of previous studies in *A. thaliana* (Villaécija-Aguilar et al., 2019), *Lotus japonicus* (Carbonnel et al., 2020) and *Brachypodium distachyon* (Meng et al., 2022). These results suggest that KAR1 has the potential to serve as an effective agent for alleviating abiotic stress during apple cultivation. Root and root hair development are regulated by the KAR−MAX2−SMAX1 signaling pathway, which affects the ethylene signaling pathway in *Lotus japonicus* (Carbonnel et al., 2020). Impairment in karrikin sensing enhances root skewing in *A. thaliana* (Swarbreck et al., 2019). Whether a similar mechanism still exists in apple needs to be further investigated.

Although KARs stimulate seed germination via the GA hormone and light signaling pathways (Nelson et al., 2009, 2010) and positively regulate plant drought resistance to regulate stomatal conductance and anthocyanin synthesis by influencing the ABA signaling pathway, in *A. thaliana* and other model plants (Li et al., 2017), Meng et al. reported that KARs negatively regulate soybean seed germination by antagonizing the biosynthesis of ABA and GA, which is different from the findings of research on *A. thaliana* (Meng et al., 2016). In the caryopses of *Avena fatua*, the sensitivity to ABA and the ratio of ABA/Gas in the coleorhiza and radicle promote dormancy (Kępczyński et al., 2024). We demonstrated that apple root development is promoted by KAR1, which mediates this process by increasing ABA and TZR and decreasing the GA3 content. The effect of KAR1 varies in different plant tissues and species. Our study is consistent with the results in *A. thaliana*, which may be attributed to differences in interspecific phylogeny and biological traits. Compared with annual woody plants, perennial woody plants may possess more complex and specialized mechanisms for adapting to environmental challenges.

The perception of KARs by KAI2 and signal transduction are ancient universal mechanisms for adapting to various plant environments. Plant *KAI2* is derived from bacterial *RsbQ* via horizontal gene transfer (HGT) before the emergence of streptophytes (Wang et al., 2022). KAI2 has a widespread phylogenetic distribution from charophytic algae to all land plants (Carbonnel et al., 2020). Our study revealed that the 130 KAI2s in different species belong to 66 families that are highly homologous to domesticated apple MdKAI2, and the sequence homology of these genes is highly consistent with their botanical classification (**Figure 2**). The transcription of *MdKAI2* is also associated with multiple stresses and has high transcriptional activity (**Supplementary Figure S3**). The widespread conservation of KAI2 implied that MdKAI2 is crucial for plant survival and has been retained long term during evolution, which is consistent with the results of Wang et al., who studied the origin and phylogenetic evolution of plant KAI2 and suggested that the evolution of plant perception of KAI2 to KLs and signal transduction reflects the systematic evolution of plants (Wang et al., 2022). Different types of KAI2 in terrestrial plants have selectivity for different forms of KAR ligands, which is a specific reflection of adaptation to the natural environment (Sun et al., 2020).

The stress resistance of several plant species has been found to be improved by KAI2. In *A. thaliana*, the anthocyanin content was significantly reduced in the *kai2* loss−of−function mutant seedling lines (Li et al., 2017), which is consistent with our findings. The biomass of *kai2* loss−of−function *A. thaliana* mutants was greater than that of wild−type seedlings with sufficient water supply, and the decrease in biomass of *kai2* mutants was greater than that of wild−type seedlings under water stress (Li et al., 2017), which was different from our results obtained for apple calli (**Figure 3E**). This difference may be related to interspecies differences in biological characteristics and the degree of stress. Our study revealed that the growth of apple calli was promoted by *MdKAI2* overexpression under normal conditions, while it was markedly inhibited under osmotic stress, cell death occurred, and the total flavonoid content significantly increased (**Figure 3A, D, E**). These findings indicate that under relatively extreme stress conditions, MdKAI2 promotes plant resistance, such as the accumulation of flavonoid compounds, by sacrificing growth to avoid being killed by stress. Interestingly, our later research revealed that biomass significantly increased under relatively weak stress (data not shown). The intensity of apple resistance mediated by MdKAI2 depends on the stress intensity. These findings indicate that the seemingly contradictory regulatory mechanisms of plant resistance and growth have reached a relatively good balance in some cases. Furthermore, the expression level of *MdKAI2* is significantly increased when it is induced by external stress, which indicates that the resistance mechanism mediated by MdKAI2 is active in the adaptation of apple to the environment.

ROS signaling significantly contributes to regulating plant growth and stress biology, and tight regulation of the redox system is highly important for balancing plant growth and stress (Ali et al., 2023). Our study confirmed that the activities of ROS−scavenging enzymes (including SOD, POD and CAT) were increased by MdKAI2 under PEG stress (**Figure 4**). The production of ROS in cells is commonly induced by stress, and ROS signaling is also a signal molecule for plant stress responses. However, excessive stress can also cause an increase in ROS and may lead to cell death. MdKAI2 may reduce the death of callus cells under relatively intense stress by increasing their ability to scavenge ROS (**Figure 3A**). Additionally, intrinsic ROS are generated and integrated networks that involve MAPK cascades, CDPK, calcineurin B−like−interacting protein kinases, ROP/RAC small GTPases, different phosphatases (PP2A, PP2C and PTPs) and several redox sensing networks, which are also consistent with the results of KEGG analyses of resistance regulation mediated by *MdKAI2* overexpression (Myers et al., 2024). Moreover, the contents of osmotic adjustment substances (including soluble protein, soluble sugars, proline, and betaine) were also increased by MdKAI2 under PEG stress. The levels of MDA and electrical conductivity decreased under PEG stress, which demonstrated that oxidative damage under stress was inhibited by MdKAI2 (**Figure 4**). Li et al. reported that cell membrane damage in *A. thaliana* was more severe under stress in *kai2* loss−of−function mutant seedlings (Li et al., 2017). Shah et al. confirmed that KAR1 treatment increased the activity of key antioxidant enzymes (including SOD, POD, CAT, and APX) in *Sapium sebiferum* and significantly reduced hydrogen peroxide, malondialdehyde, and electrolyte leakage under abiotic stresses (Shah et al., 2020). These findings also provide important physiological evidence that plant stress resistance is enhanced by KAI2−KAR signaling.

The results of the omics analysis provide an important theoretical basis for exploring the regulatory mechanism of target genes. MdKAI2 significantly influenced the transcript levels of several TFs involved in the resistance response, including *MYB*, *bHLH*, *AP2−EREBP*, *NAC*, *ABI3VP1*, *MADS*, and *WRKY* (**Figure 5E**). MYB TFs can improve plant resistance by regulating anthocyanin biosynthesis (Geng et al., 2020). In this study, the greatest number of 9 differentially expressed *MYB* TFs were detected, which is consistent with the finding that the flavonoid content increased in the *MdKAI2* transgenic lines (**Figure 3D**). The ABA signaling pathway is involved in the regulation of plant resistance by KAI2 (Li et al., 2017; Shah et al., 2020). ABI3VP1 (Zhao et al., 2020a) and WRKY (Zhao et al., 2019) are both involved in the ABA signaling response, and specific WRKY transcription factors might be conserved mediators of KAI2−dependent signaling. We found that the contents of the endogenous hormones ABA and TZR increased and that of GA3 decreased in response to MdKAI2, which confirmed that KAR1−MdKAI2 is involved in the regulation of apple hormones (**Figures 1D–F**), that two and one *ABI3VP1* members were up− and downregulated, respectively, and that two *WRKY* members were downregulated by MdKAI2 (**Figure 5E**). MADS, a negative regulatory factor for plant drought resistance (Zhao et al., 2020b), was significantly downregulated (**Figure 5E**), which implied that MdKAI2 may increase apple resistance by negatively regulating the MADS expression level. In addition, bHLH, AP2−EREBP, and NAC TFs play important roles (Zhao et al., 2020c; Zhang et al., 2019; Jia et al., 2019). The mechanisms by which TFs respond to MdKAI2 and regulate the ABA signaling pathway need to be further studied.

According to the results of the KEGG analyses, BR biosynthesis was negatively regulated by MdKAI2 (**Figure 6B**). BRs play crucial roles in diverse types of plant abiotic stress resistance and serve as negative regulators of plant pattern−triggered immunity (PTI) because they lead to the sacrifice of defense at the cost of increased growth (Yu et al., 2018). Immune resistance in plants is similar to reproductive growth (Mondragón-Palomino et al., 2017), and both involve specific mechanisms through which plants themselves can respond to exogenous substance signals. This process is highly similar to that of KAR1−MdKAI2 signaling, and the endogenous receptor MdKAI2 is capable of specifically recognizing exogenous KAR1. We also found that plant−pathogen interaction pathways were enhanced by MdKAI2. This might be one of the reasons why the biosynthesis of BRs is negatively regulated by MdKAI2 (**Figure 6**). Whether KAR1−MdKAI2 negatively regulates BR signaling and augments resistance by reducing growth under relatively extreme stress (**Figure 3A, E**) requires further investigation. The results of the RNA−Seq and metabolome analyses confirmed that multiple stress resistance pathways involving hormone signal transduction, the metabolism of amino acids and lipids, the biosynthesis of defense−related secondary metabolites, and the MAPK signaling pathway were also markedly regulated by MdKAI2 (**Figures 6**−**9**). At present, there are few reports on the transcriptional and metabolic mechanisms of KAI2 in the regulation of plant resistance. Our results provide new insights into plant resistance mediated by KAR1−MdKAI2.

Furthermore, L−ectoine, a soluble substance that endows halophilic bacteria with the ability to survive in high−salt environments, is upregulated 10−fold by MdKAI2 and has been shown to significantly increase tolerance to high osmolarity in tomato (Moghaieb et al., 2011) and tobacco (Nakayama et al., 2000). At present, plant endogenous L−ectoine has not yet been reported. We excluded the possibility that the strains originated from microorganisms, including *Agrobacterium*, because the transgenic calli were sterile. Moreover, the level of L−ectoine was significantly greater in the *MdKAI2* transgenic lines than in the CT lines, and both callus lines were generated via *Agrobacterium*. Whether plants possess endogenous L−ectoine and whether this process is mediated by MdKAI2 warrant further study. It would be an exciting discovery if that is the case.

## Conclusion

KAR1 significantly enhances apple root development and promotes the upregulation of the hormones ABA and TZR and the downregulation of GA3 in apple roots. *MdKAI2* can be markedly upregulated in response to osmotic stress. MdKAI2 enhances apple resistance through regulating growth and development as well as the accumulation of multiple resistance substances and mainly regulates multiple pathways related to apple stress resistance, including hormone signal transduction, the metabolism of amino acids and lipids, the biosynthesis of defense−related secondary metabolites, and signaling pathways. MdKAI2 positively regulates fatty acids, esters, alcohols, and terpenoids but negatively regulates the metabolites of amino acid derivatives and amides, and the MAPK signaling pathway may mediate these processes.

## Materials and methods

### KAR1 treatment of apple tissue culture–generated seedlings and determination of plant hormones

Gala tissue culture seedlings (Hu et al., 2020) were subcultured in MS media (0.5 mg·L^-1^ 6−BA, 0.2 mg·L^-1^ IAA) at 24°C with a photoperiod of 16 hours of light and 8 hours of darkness. A rooting−inducing assay was conducted in ½ MS media supplemented with 0.1 mg·L^-1^ IAA or KAR1 (CAS: 857054−02−5) at different concentrations. The culture conditions were the same as those for the subcultures described above, and photographs were taken after treatment for 58 days. The contents of ABA, GA3 and TZR plant hormones in apple seedling roots subcultured for 4 days in MS media with KAR1 concentrations of 0, 1 nM, 5 nM, 50 nM, and 500 nM were determined via the LC−MS method (Hashiguchi et al., 2021).

### Protein bioinformatics and evolutionary relationship analysis of MdKAI2

Physical and chemical property analyses were conducted via the ProtParam online program (https://web.ExPASy.org/protparam/). The protein secondary structure of MdKAI2 was determined via the SOPMA online program (https://npsa-pbil.ibcp.fr/cgi-bin/npsa_automat.pl?page=npsa_sopma.html). Protein transmembrane structure analysis was conducted via the TMHMM online program (https://services.healthtech.dtu.dk/services/TMHMM-2.0/). Protein hydrophilicity analysis was conducted via the ProtScale online program (https://web.ExPASy.org/protscale/). Protein motif analysis was conducted via the MEME online program (https://meme-suite.org/meme/tools/meme). The promoter region of the 2000 nucleotide sequence was retrieved upstream of the ATG translation start codon of the *MdKAI2* gene.

The *AtKAI2* coding sequence in *A. thaliana* was acquired from the TAIR (The Arabidopsis Information Resource) database with the gene ID AT4G37470.1, and the *MdKAI2* coding sequence in *M. domestica* was acquired from the GDR (Genome Database for Rosaceae) database with the gene ID MD02G1181300. A total of 129 amino acid−coding sequences of orthologous genes highly consistent with those of MdKAI2 were aligned via GenBank, and one representative coding sequence per species was retrieved, together with the amino acid−coding sequences of AtKAI2 and MdKAI2, for analysis of the evolutionary relationship. A phylogenetic tree was constructed via MEGA 7 software, the neighbor−joining method was used to evaluate the evolutionary relationships, and the evolutionary distances were calculated via Poisson correction.

### Construction of genetic transformation lines of *MdKAI2* in apple calli

The primers used for amplification of the open reading frame (ORF) of *MdKAI2* are shown in **Supplementary Table S4**. The nucleotide fragment of the coding sequence was inserted into the pBI121 vector for the construction of a binary recombinant pBI121−*CaMV 35S*::*MdKAI2*−*GFP* overexpression vector, which was subsequently transfected into Orin apple calli (Wang et al., 2021) generated via *Agrobacterium*. An empty vector without the coding sequence (pBI121−*CaMV 35S*::*GFP*) was also constructed and used as a control. The positive transgenic calli were screened on MS media supplemented with 15 g·L^-1^ agar, 1.5 mg·L^-1^ 2,4−D, 0.5 mg·L^-1^ 6−BA and 50 mg·L^-1^ kanamycin A and were identified via both qRT−PCR and western blotting. Transgenic Orin calli were cultured in the dark at 24°C and subcultured every 15 days.

### PEG treatment of apple calli

Orin transgenic calli were inoculated on PEG−infused plates after subculturing for 10 days. The PEG−infused plant system was constructed according to the method of Verslues et al. (2006). Because PEG prevents polymerization of the agar, the PEG−containing overlay solution was added on top of the solidified MS medium. PEG6000 was dissolved in liquid ½ MS media supplemented with 2 mM 2−morpholinoethanesulfonic acid (MES) without agar at a 20% final mass ratio of PEG. Except for MES, which was sterilized by filtration, the PEG overlay solution was autoclaved before use. The solidified MS media were prepared with 15 g·L^-1^ agar, 1.5 mg·L^-1^ 2,4−D and 0.5 mg·L^-1^ 6−BA, and the pH was adjusted to 6.0. The remaining PEG overlay solution was removed after infusion and infiltration into solidified MS media for 12 hours.

### Determination of physical and chemical indicators

The water content of the callus was calculated as the ratio of the water mass to the fresh weight. The biomass of the callus was measured by weighing the fresh weight after culture for 10 days. SOD activity was measured via the nitrogen blue tetrazole (NBT) photochemical reduction method according to the method described by Chang et al. (2022). POD activity was measured via the guaiacol oxidation method (Wang et al., 2019). CAT activity was measured via the ammonium molybdate method (Chang et al., 2022). The MDA, total soluble sugar and proline levels in the calli were measured according to the methods described by Chang et al. (2022). The total soluble protein content was analyzed via a Coomassie brilliant blue protein analysis kit (Jining, China). The relative electrical conductivity was measured according to the method described by Li et al. (2019). The glycine betaine content was measured according to the method described by Gupta et al. (2014).

### Omics analysis of transgenic apple calli

Orin transgenic calli of *CaMV 35S*::*MdKAI2*−*GFP* (OE) were selected for RNA−Seq analysis, and transgenic calli of *CaMV 35S*::*GFP* (CT) were used for the control group. Three independent callus lines from each group were used for the repeats. mRNA extraction, cDNA library construction, next−generation sequencing and annotation of the transcripts were carried out by the BGI Company (China). The reference annotation information of the transcripts was derived from the genome data of *M. domestica* (Daccord et al., 2017) published in the GDR (https://www.rosaceae.org/) database. Metabolomic analysis of the abovementioned transgenic Orin calli was carried out via high−performance liquid chromatography−mass spectrometry (HPLC−MS) by the BGI Company (China).

### Statistical analysis

The root projection area was calculated via ImageJ software (version 1.52 V), and the total root length was calculated via WinRHIZO software (version 2013). The evolutionary relationships among orthologous proteins of MdKAI2 were analyzed and plotted via Mega 7 software (version 7.0.26). The 2^-ΔΔCT^ method was used to analyze the qRT−PCR data, and the primers used are shown in **Supplementary Table S4**. PCA and PLS−DA were performed via Simca software (version 14.1). Correlation analysis, volcano plots and cluster heatmaps were generated via Origin Pro software (version 2021). Transcription factors were annotated via PlantTFDB (https://planttfdb.gao-lab.org/). Diagrams of the KEGG and correlation analyses of the DAMs or DEGs were generated via the OmicShare online program (https://www.omicshare.com/), and bubble and column charts were drawn via Microsoft Excel software (version 2019).

One−way analysis of variance (ANOVA) and multiple comparisons via Fisher’s least significant difference (LSD) test were performed via IBM SPSS Statistics software (version 23). The homoscedasticity and normal distribution of the data were tested via Levene’s test and the Shapiro−Wilk test, respectively. Student’s t test with paired and two−tailed distributions for pairwise comparisons was conducted via Microsoft Excel software (version 2019). The column charts were drawn via GraphPad Prism software (version 9.5.0).

## Data Availability

All data supporting the findings of this study are available within its **Supplementary Information** or within the online repository The National Center for Biotechnology Information BioProject PRJNA1137529. The materials used during the current study are available from the corresponding author upon reasonable request.

## References

[B1] Ali S. Tyagi A. Bae H. (2023). ROS interplay between plant growth and stress biology: Challenges and future perspectives. Plant Physiol. Bioch. 203, 108032. doi: 10.1016/j.plaphy.2023.108032 37757722

[B2] Carbonnel S. Das D. Varshney K. Kolodziej M. C. Villaécija-Aguilar J. A. Gutjahr C. (2020). The karrikin signaling regulator SMAX1 controls Lotus japonicus root and root hair development by suppressing ethylene biosynthesis. Proc. Natl. Acad. Sci. U.S.A. 117, 21757–21765. doi: 10.1073/pnas.2006111117 32817510 PMC7474694

[B3] Chang T. Xi Q. Wei X. Xu L. Wang Q. Fu J.. (2022). Rhythmical redox homeostasis can be restored by exogenous melatonin in hulless barley (*Hordeum vulgare* L.var. nudum) under cold stress. Environ. Exp. Bot. 194, 104756. doi: 10.1016/j.envexpbot.2021.104756

[B4] Daccord N. Celton J. M. Linsmith G. Becker C. Choisne N. Schijlen E.. (2017). High-quality *de novo* assembly of the apple genome and methylome dynamics of early fruit development. Nat. Genet. 49, 1099–1106. doi: 10.1038/ng.3886 28581499

[B5] Flematti G. R. Dixon K. W. Smith S. M. (2015). What are karrikins and how were they ‘discovered’ by plants? BMC Biol. 13, 108. doi: 10.1186/s12915-015-0219-0 26689715 PMC4687367

[B6] Flematti G. R. Ghisalberti E. L. Dixon K. W. Trengove R. D. (2004). A compound from smoke that promotes seed germination. Science 305, 977. doi: 10.1126/science.1099944 15247439

[B7] Geng D. Shen X. Xie Y. Yang Y. Bian R. Gao Y.. (2020). Regulation of phenylpropanoid biosynthesis by MdMYB88 and MdMYB124 contributes to pathogen and drought resistance in apple. Hortic. Res. 7, 102. doi: 10.1038/s41438-020-0324-2 32637130 PMC7327078

[B8] Gupta N. Thind S. K. Bains N. S. (2014). Glycine betaine application modifies biochemical attributes of osmotic adjustment in drought stressed wheat. Plant Growth Regul. 72, 221–228. doi: 10.1007/s10725-013-9853-0

[B9] Hashiguchi T. Hashiguchi M. Tanaka H. Fukushima K. Gondo T. Akashi R. (2021). Quantitative analysis of seven plant hormones in *Lotus japonicus* using standard addition method. PloS One 16, e0247276. doi: 10.1371/journal.pone.0247276 33600422 PMC7891737

[B10] Hu D. G. Sun C. H. Zhang Q. Y. Gu K. D. Hao Y. J. (2020). The basic helix-loop-helix transcription factor MdbHLH3 modulates leaf senescence in apple via the regulation of *dehydratase-enolase-phosphatase complex 1* . Hortic. Res. 7, 50. doi: 10.1038/s41438-020-0273-9 32257236 PMC7109056

[B11] Jia D. Jiang Q. van Nocker S. Gong X. Ma F. (2019). An apple (*Malus domestica*) NAC transcription factor enhances drought tolerance in transgenic apple plants. Plant Physiol. Bioch. 139, 504–512. doi: 10.1016/j.plaphy.2019.04.011 31015089

[B12] Kępczyński J. Dziurka M. Wójcik A. (2024). KAR1-induced dormancy release in Avena fatua caryopses involves reduction of caryopsis sensitivity to ABA and ABA/GAs ratio in coleorhiza and radicle. Planta 259, 126. doi: 10.1007/s00425-024-04387-1 38635035 PMC11026216

[B13] Khosla A. Morffy N. Li Q. Faure L. Chang S. H. Yao J.. (2020). Structure-function analysis of SMAX1 reveals domains that mediate its karrikin-induced proteolysis and interaction with the receptor KAI2. Plant Cell. 32, 2639–2659. doi: 10.1105/tpc.19.00752 32434855 PMC7401016

[B14] Li H. Mo Y. L. Cui Q. Yang X. Z. Guo Y. L. Wei C. H.. (2019). Transcriptomic and physiological analyses reveal drought adaptation strategies in drought-tolerant and -susceptible watermelon genotypes. Plant Sci. 278, 32–43. doi: 10.1016/j.plantsci.2018.10.016 30471727

[B15] Li W. Nguyen K. H. Chu H. D. Ha C. V. Watanabe Y. Osakabe Y.. (2017). The karrikin receptor KAI2 promotes drought resistance in *Arabidopsis thaliana* . PloS Genet. 13, e1007076. doi: 10.1371/journal.pgen.1007076 29131815 PMC5703579

[B16] Ludwig A. A. Saitoh H. Felix G. Freymark G. Miersch O. Wasternack C.. (2005). Ethylene-mediated cross-talk between calcium-dependent protein kinase and MAPK signaling controls stress responses in plants. Proc. Natl. Acad. Sci. U.S.A. 102, 10736–10741. doi: 10.1073/pnas.0502954102 16027369 PMC1176231

[B17] Meng Y. Chen F. Shuai H. Luo X. Ding J. Tang S.. (2016). Karrikins delay soybean seed germination by mediating abscisic acid and gibberellin biogenesis under shaded conditions. Sci. Rep. 6, 22073. doi: 10.1038/srep22073 26902640 PMC4763256

[B18] Meng Y. Varshney K. Incze N. Badics E. Kamran M. Davies S. F.. (2022). *KARRIKIN INSENSITIVE2* regulates leaf development, root system architecture and arbuscular-mycorrhizal symbiosis in *Brachypodium distachyon* . Plant J. 109, 1559–1574. doi: 10.1111/tpj.v109.6 34953105

[B19] Mizuno Y. Komatsu A. Shimazaki S. Naramoto S. Inoue K. Xie X.. (2021). Major components of the KARRIKIN INSENSITIVE2-dependent signaling pathway are conserved in the liverwort *Marchantia polymorpha* . Plant Cell. 33, 2395–2411. doi: 10.1093/plcell/koab106 33839776 PMC8364241

[B20] Moghaieb R. E. Nakamura A. Saneoka H. Fujita K. (2011). Evaluation of salt tolerance in ectoine-transgenic tomato plants (*Lycopersicon esculentum*) in terms of photosynthesis, osmotic adjustment, and carbon partitioning. GM Crops 2, 58–65. doi: 10.4161/gmcr.2.1.15831 21844699

[B21] Mondragón-Palomino M. John-Arputharaj A. Pallmann M. Dresselhaus T. (2017). Similarities between reproductive and immune pistil transcriptomes of *Arabidopsis* species. Plant Physiol. 174, 1559–1575. doi: 10.1104/pp.17.00390 28483878 PMC5490908

[B22] Myers R. J. Peláez-Vico M.Á. Fichman Y. (2024). Functional analysis of reactive oxygen species-driven stress systemic signalling, interplay and acclimation. Plant Cell Environ. 47, 2842–2851. doi: 10.1111/pce.14894 38515255

[B23] Nakayama H. Yoshida K. Ono H. Murooka Y. Shinmyo A. (2000). Ectoine, the compatible solute of *Halomonas elongata*, confers hyperosmotic tolerance in cultured tobacco cells. Plant Physiol. 122, 1239–1247. doi: 10.1104/pp.122.4.1239 10759521 PMC58960

[B24] Nasir F. Li W. Q. Tran L. S. P. Tian C. J. (2020). Does karrikin signaling shape the rhizomicrobiome via the strigolactone biosynthetic pathway? Trends Plant Sci. 25, 1184–1187. doi: 10.1016/j.tplants.2020.08.005 32888808

[B25] Nelson D. C. Flematti G. R. Riseborough J. A. Ghisalberti E. L. Dixon K. W. Smith S. M. (2010). Karrikins enhance light responses during germination and seedling development in *Arabidopsis thaliana* . Proc. Natl. Acad. Sci. U.S.A. 107, 7095–7100. doi: 10.1073/pnas.0911635107 20351290 PMC2872431

[B26] Nelson D. C. Riseborough J. A. Flematti G. R. Stevens J. Ghisalberti E. L. Dixon K. W.. (2009). Karrikins, discovered in smoke trigger *Arabidopsis* seed germination by a mechanism requiring gibberellic acid synthesis and light. Plant Physiol. 149, 863–873. doi: 10.1104/pp.108.131516 19074625 PMC2633839

[B27] Shah F. A. Wei X. Wang Q. Liu W. Wang D. Yao Y.. (2020). Karrikin improves osmotic and salt stress tolerance via the regulation of the redox homeostasis in the oil plant *Sapium sebiferum* . Front. Plant Sci. 11, 216. doi: 10.3389/fpls.2020.00216 32265947 PMC7105677

[B28] Sun Y. K. Yao J. Scaffidi A. Melville K. T. Davies S. F. Bond C. S.. (2020). Divergent receptor proteins confer responses to different karrikins in two ephemeral weeds. Nat. Commun. 11, 1264. doi: 10.1038/s41467-020-14991-w 32152287 PMC7062792

[B29] Swarbreck S. M. Guerringue Y. Matthus E. Jamieson F. J. C. Davies J. M. (2019). Impairment in karrikin but not strigolactone sensing enhances root skewing in *Arabidopsis thaliana* . Plant J. 98, 607–621. doi: 10.1111/tpj.2019.98.issue-4 30659713 PMC6563046

[B30] Verslues P. E. Agarwal M. Katiyar-Agarwal S. Zhu J. Zhu J. K. (2006). Methods and concepts in quantifying resistance to drought, salt and freezing, abiotic stresses that affect plant water status. Plant J. 45, 523–539. doi: 10.1111/j.1365-313X.2005.02593.x 16441347

[B31] Villaécija-Aguilar J. A. Hamon-Josse M. Carbonnel S. Kretschmar A. Schmidt C. Dawid C.. (2019). SMAX1/SMXL2 regulate root and root hair development downstream of KAI2-mediated signalling in *Arabidopsis* . PloS Genet. 15, e1008327. doi: 10.1371/journal.pgen.1008327 31465451 PMC6738646

[B32] Wang X. Q. Du G. D. Lu X. F. Ma H. Y. Lyu D. G. Zhang H.. (2019). Characteristics of mitochondrial membrane functions and antioxidant enzyme activities in strawberry roots under exogenous phenolic acid stress. Sci. Hortic. 248, 89–97. doi: 10.1016/j.scienta.2018.12.051

[B33] Wang Q. Smith S. M. Huang J. (2022). Origins of strigolactone and karrikin signaling in plants. Trends Plant Sci. 27, 450–459. doi: 10.1016/j.tplants.2021.11.009 34876337

[B34] Wang X. Xu L. Liu X. Xin L. Wu S. Chen X. (2021). Development of potent promoters that drive the efficient expression of genes in apple protoplasts. Hortic. Res. 8, 211. doi: 10.1038/s41438-021-00646-4 34593780 PMC8484340

[B35] Waters M. T. Scaffidi A. Moulin S. L. Y. Sun Y. K. Flematti G. R. Smith S. M. (2015). A *Selaginella moellendorffii* ortholog of KARRIKIN INSENSITIVE2 functions in *Arabidopsis* development but cannot mediate responses to karrikins or strigolactones. Plant Cell. 27, 1925–1944. doi: 10.1105/tpc.15.00146 26175507 PMC4531350

[B36] Yu M. H. Zhao Z. Z. He J. X. (2018). Brassinosteroid signaling in plant-microbe interactions. Int. J. Mol. Sci. 19, 4091. doi: 10.3390/ijms19124091 30563020 PMC6320871

[B37] Zhang Y. L. Zhang C. L. Wang G. L. Wang Y. X. Qi C. H. You C. X.. (2019). Apple AP2/EREBP transcription factor MdSHINE2 confers drought resistance by regulating wax biosynthesis. Planta 249, 1627–1643. doi: 10.1007/s00425-019-03115-4 30826884

[B38] Zhao Q. Fan Z. Qiu L. Che Q. Wang T. Li Y.. (2020c). MdbHLH130, an apple bHLH transcription factor, confers water stress resistance by regulating stomatal closure and ROS homeostasis in transgenic tobacco. Front. Plant Sci. 11, 543696. doi: 10.3389/fpls.2020.543696 33163009 PMC7581937

[B39] Zhao P. X. Miao Z. Q. Zhang J. Chen S. Y. Liu Q. Q. Xiang C. B. (2020b). *Arabidopsis* MADS-box factor AGL16 negatively regulates drought resistance via stomatal density and stomatal movement. J. Exp. Bot. 71, 6092–6106. doi: 10.1093/jxb/eraa303 32594177

[B40] Zhao H. Nie K. Zhou H. Yan X. Zhan Q. Zheng Y.. (2020a). ABI5 modulates seed germination via feedback regulation of the expression of the PYR/PYL/RCAR ABA receptor genes. New Phytol. 228, 596–608. doi: 10.1111/nph.v228.2 32473058

[B41] Zhao X. Y. Qi C. H. Jiang H. You C. X. Guan Q. M. Ma F. W.. (2019). The MdWRKY31 transcription factor binds to the MdRAV1 promoter to mediate ABA sensitivity. Hortic. Res. 6, 66. doi: 10.1038/s41438-019-0147-1 31231524 PMC6544635

