# Supplementary material for: Identification of osmotic stress resistance mediated by MdKAI2 in apple
Source: Front Plant Sci. 2024 Dec 5;15:1467034. doi: 10.3389/fpls.2024.1467034 (PMC11655239; doi:10.3389/fpls.2024.1467034)
Supplement: Supplementary file 1 [file DataSheet1.docx]

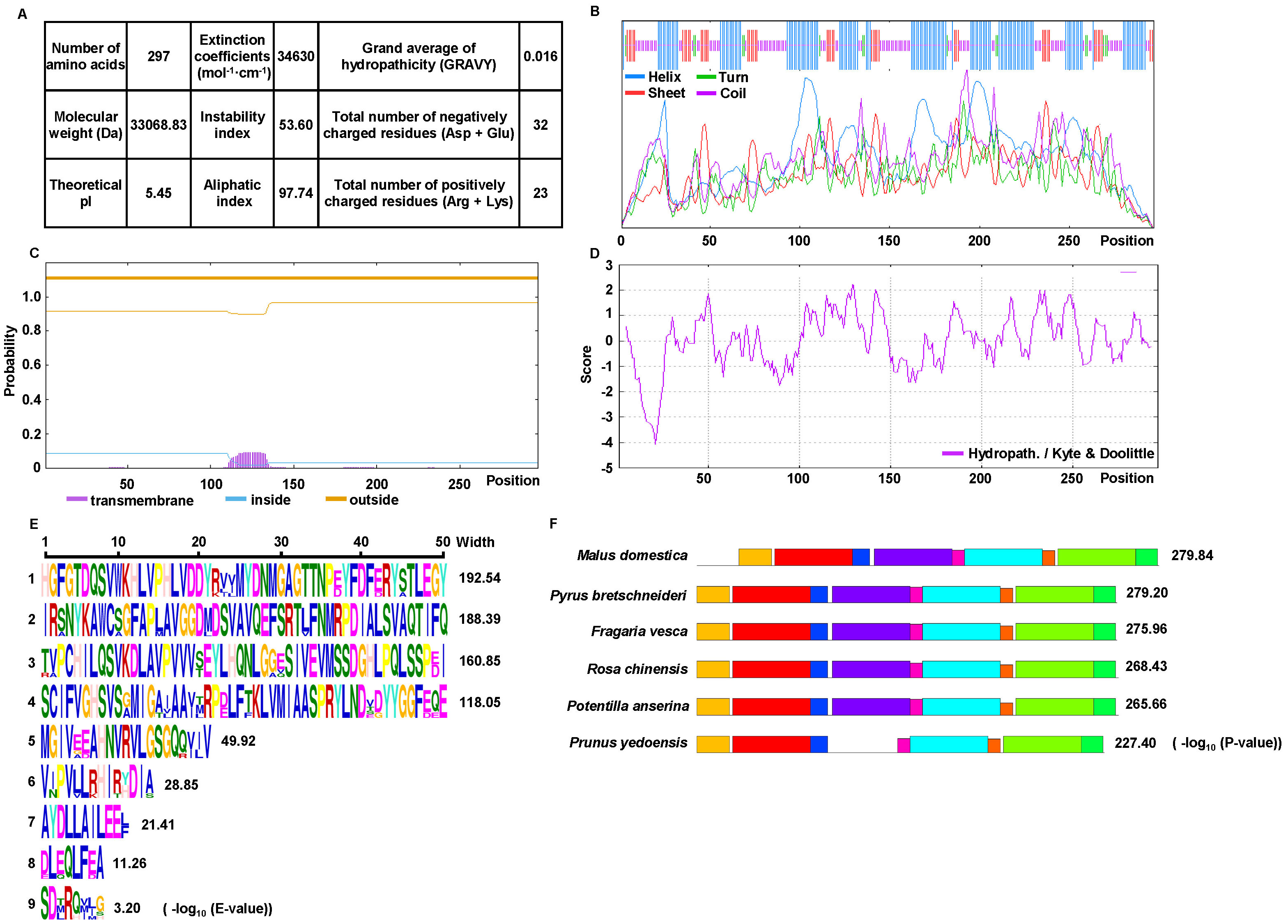


**Figure S1** Bioinformatic analysis of the MdKAI2 protein. Prediction of the physical and chemical properties (**A**), secondary structure (**B**), transmembrane structure (**C**), hydrophilicity (**D**) and motif (**E, F**) of MdKAI2. Physical and chemical property predictions were conducted via the ProtParam online program. Protein secondary structure prediction was conducted via the SOPMA online program. Protein transmembrane structure prediction was conducted via the TMHMM online program. Protein hydrophilicity prediction was conducted via the ProtScale online program. Protein motif prediction was conducted via the motif discovery tool of the MEME online program.


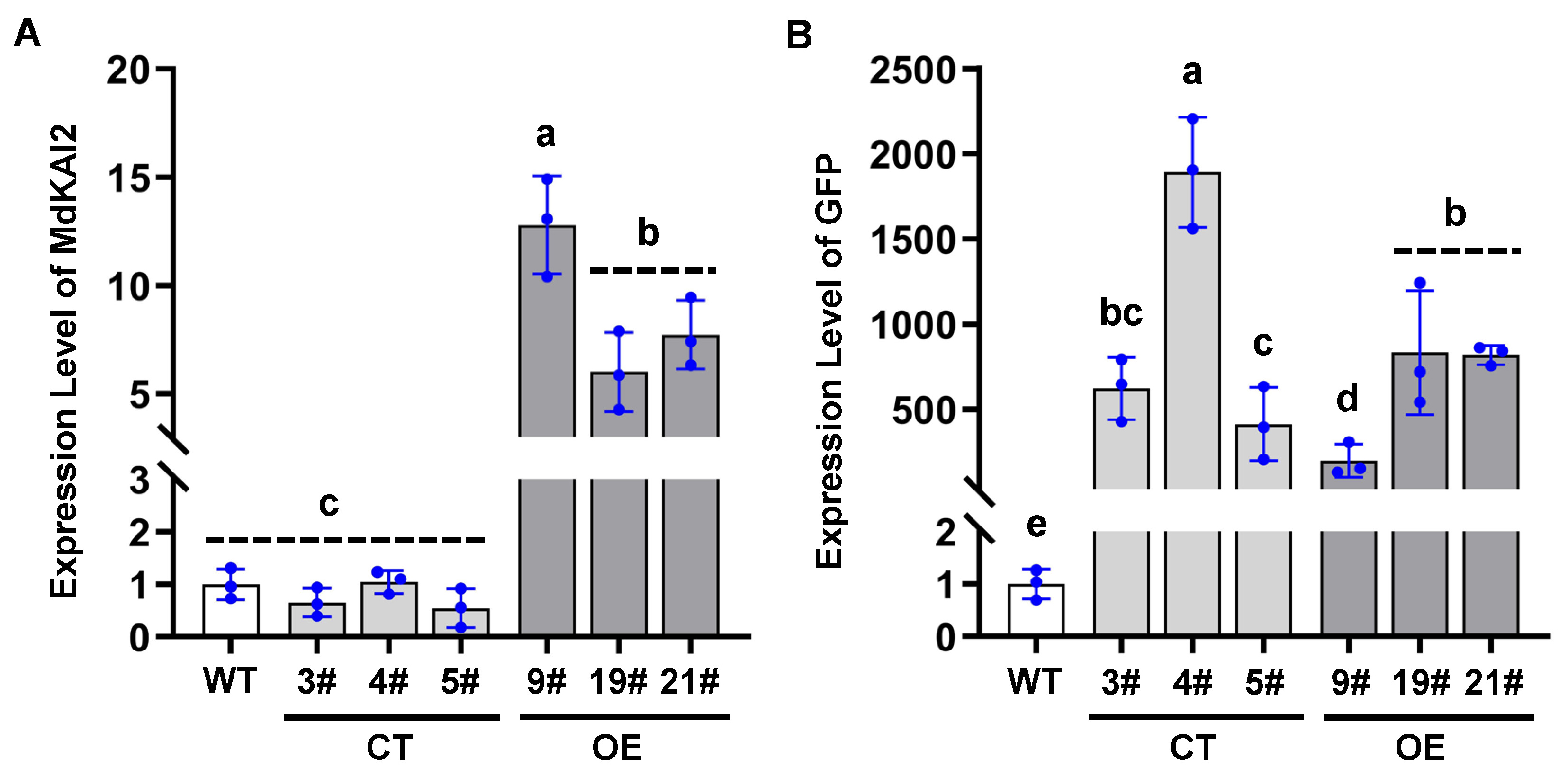


**Figure S2** Identification of the *MdKAI2* transcript in the overexpressing calli. The relative expression levels of *MdKAI2* (A) and *GFP* (B) were analyzed via qRT‒PCR. Three independent transformed callus lines were selected for testing. The CT lines transformed with the recombinant pBI121‒*CaMV 35S*::*GFP* vector were labeled #3, #4 and #5. The OE lines transformed with the recombinant pBI121‒*CaMV 35S*::*MdKAI2*‒*GFP* vector are labeled #9, #19 and #21. One‒way ANOVA and multiple comparisons via Fisher’s LSD test were performed. Three independent experiments were conducted, and the individual points for each biological replicate are marked in each column. The different characters at the top of the columns indicate significant differences (p<0.05). The homoscedasticity and normal distribution of the data were confirmed by Levene's test and the Shapiro‒Wilk test, respectively.


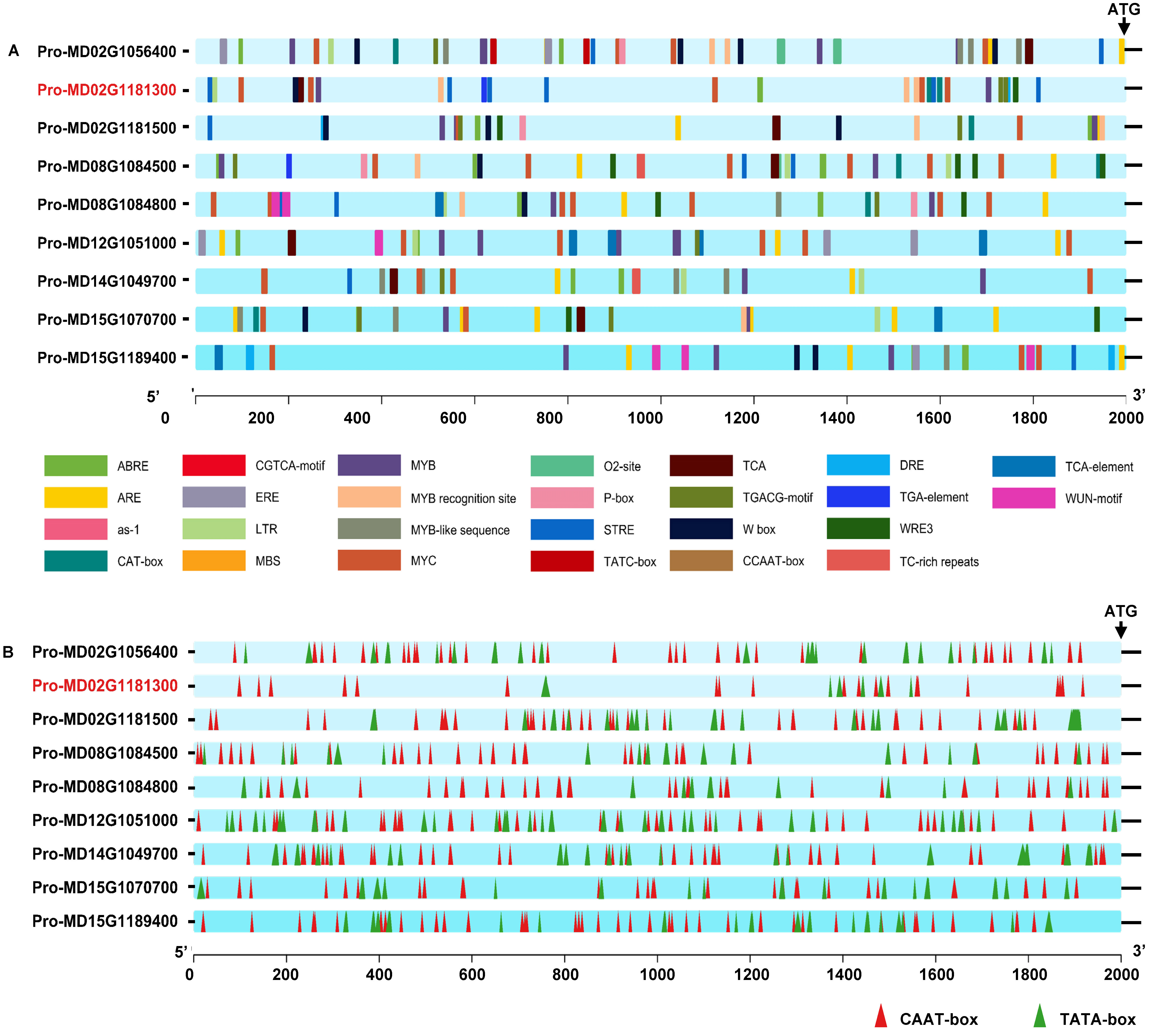


**Figure S3** Cis‒element prediction of *MdAKI2* paralogous genes in apple. Using the PlanrCare online program, the candidate promoter region of *MdKAI2*, which is 2000 nucleotides upstream of the ATG translation start codon of the apple *MdKAI2* (MD02G1181300) gene, was used for cis‒element prediction. Except for the predicted CAAT box and TATA boxes (**B**), the other cis‒elements are shown in **A**.


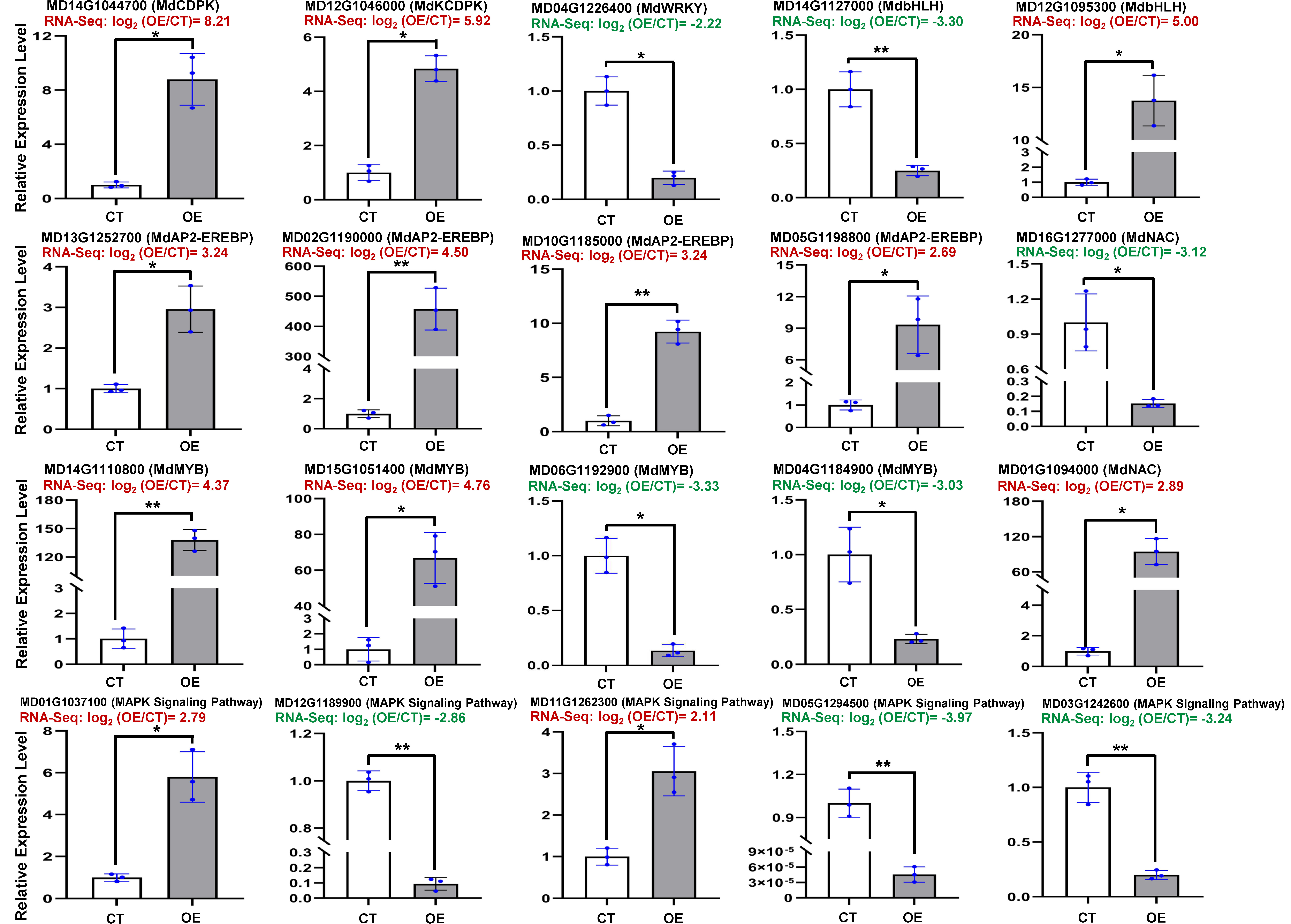


**Figure S4 Verification of functional genes associated with plant resistance via qRT‒PCR.**

Twenty key functional genes associated with plant resistance were selected for qRT‒PCR verification, including *MdbHLH*, *MdAP2*‒*EREBP*, *MdMYB*, *MdNAC*, *MdCDPK*, *MdWRKY*, and MAPK signaling related genes. Student’s t test with paired and two‒tailed distribution for pairwise comparisons, and the asterisks indicate a significant difference (*p<0.05, **p<0.01).
