# Supplementary material for: Identification of osmotic stress resistance mediated by MdKAI2 in apple
Source: Front Plant Sci. 2024 Dec 5;15:1467034. doi: 10.3389/fpls.2024.1467034 (PMC11655239; doi:10.3389/fpls.2024.1467034)
Supplement: Supplementary file 2 [file DataSheet2.docx]

**Supplementary information**

**Table S1 Information on 37 annotated metabolites with different abundances**

| **Code Number** | **Name** |
| --- | --- |
| **M**‒**1** | 19(R)-Hydroxy prostaglandin E1 |
| **M**‒**2** | Cucurbitacin O |
| **M**‒**3** | 15(R)-Lipoxin A4 |
| **M**‒**4** | 4-tert-Butylbenzo-15-crown-5 |
| **M**‒**5** | 3-(4-fluorophenoxy)-1-(1,4-thiazinan-4-yl)propan-1-one |
| **M**‒**6** | methyl 2-(acetylamino)-4,5-dimethoxybenzoate |
| **M**‒**7** | Estriol |
| **M**‒**8** | Capsiate |
| **M**‒**9** | 2,3-dinor-8-iso Prostaglandin F2α |
| **M**‒**10** | 2,3-Dinor prostaglandin E1 |
| **M**‒**11** | Dihydrocucurbitacin F |
| **M**‒**12** | L-Ectoine |
| **M**‒**13** | 4-(beta-D-Glucopyranosyloxy)benzyl 4,5-dideoxy-3-C-methylpentonate |
| **M**‒**14** | (9R,10R)-10-hydroxy-8,8-dimethyl-9-{[(2S,3R,4S,5S,6R)-3,4,5-trihydroxy-6-(hydroxymethyl)oxan-2-yl]oxy}-2H,8H,9H,10H-pyrano[2,3-h]chromen-2-one |
| **M**‒**15** | PI(18:3(9Z,12Z,15Z)/0:0) |
| **M**‒**16** | PI(18:3(6Z,9Z,12Z)/0:0) |
| **M**‒**17** | N-Acetyltyramine |
| **M**‒**18** | Pfaffic acid |
| **M**‒**19** | Glabrolide |
| **M**‒**20** | Quillaic acid |
| **M**‒**21** | 8-(2,4-Dimethoxyphenyl)-2-(3-fluorobenzoyl)-1,3,4,12a-tetrahydropyrazino[2,1-c][1,4]benzodiazepine-6,12(2H,11H)-dione |
| **M**‒**22** | N-{4-[(2R,3R)-3-(Hydroxymethyl)-4-methyl-5-oxo-2-morpholinyl]phenyl}-5-methyl-1,2-oxazole-3-carboxamide |
| **M**‒**23** | 2-(Glutathion-S-yl)-2-methylbut-3-en-1-ol |
| **M**‒**24** | 1,4:3,6-Dianhydro-2-deoxy-5-O-[(4-methoxyphenyl)carbamoyl]-2-[(propylsulfonyl)amino]-D-glucitol |
| **M**‒**25** | Lignicol |
| **M**‒**26** | PA(12:0/15:1(9Z)) |
| **M**‒**27** | N-Succinyl-L-citrulline |
| **M**‒**28** | 3-(tert-butyl)-1-(4-fluorobenzyl)-N-(4-fluorophenyl)-1H-pyrazole-5-carboxamide |
| **M**‒**29** | 1-(2-{[benzyl(methylsulfonyl)amino]methyl}phenyl)-4-piperidinecarboxylic acid |
| **M**‒**30** | 1-(4-methyl-2-nitrophenoxy)-3-[4-(3-phenyl-1,2,4-thiadiazol-5-yl)piperazino]propan-2-ol |
| **M**‒**31** | Ancymidol |
| **M**‒**32** | Pipecolic acid |
| **M**‒**33** | N'1-benzylidene-2-{3-[(4-methyl-1,3-thiazol-2-yl)methyl]-4-oxo-3,4-dihydrophthalazin-1-yl}ethanohydrazide |
| **M**‒**34** | Santiaguine |
| **M**‒**35** | (4-methylphenyl)[4-(7-methylthieno[3,2-d]pyrimidin-4-yl)piperazino]methanone |
| **M**‒**36** | 2-[(2S,3R,4S,5R)-5-(Aminomethyl)-3,4-dihydroxytetrahydro-2-furanyl]-N-(4-methoxybenzyl)acetamide |
| **M**‒**37** | (15Z)-9,12,13-Trihydroxy-15-octadecenoic acid |

Notes: The metabolite IDs represent the names of the metabolites in Figure 9.

**Table S2 Information on *MdKAI2* and DEGs involved in MAPK signaling pathways**

| **Code number** | **Gene ID** | **NCBI annotation** | **E-value** | **Per. Ident (%)** |
| --- | --- | --- | --- | --- |
| **G**‒**1** | MD02G1274600 | Rust Resistance Kinase Lr10-like [*Malus domestica*] | 0 | 100.00 |
| **G**‒**2** | MD15G1212000 | Protein Phosphatase 2C 77 [*Malus domestica*] | 0 | 100.00 |
| **G**‒**3** | MD00G1101900 | Cysteine-rich Receptor-like Protein Kinase 25 [*Malus domestica*] | 0 | 100.00 |
| **G**‒**4** | MD11G1010600 | Serine/threonine-protein Phosphatase PP2A Catalytic Subunit [*Malus domestica*] | 0 | 100.00 |
| **G**‒**5** | MD15G1437400 | probable Leucine-rich Repeat Receptor-like Protein Kinase At1g35710 [*Malus domestica*] | 0 | 100.00 |
| **G**‒**6** | MD05G1294500 | probable LRR Receptor-like Serine/threonine-protein Kinase At1g07650 [*Malus domestica*] | 2.0×10^-175^ | 98.37 |
| **G**‒**7** | MD14G1127000 | Transcription Factor MYC2-like [*Malus sylvestris*] | 0 | 100.00 |
| **G**‒**8** | MD03G1242600 | protein Light-dependent Short Hypocotyls 10 [*Malus domestica*] | 1.0×10^-136^ | 100.00 |
| **G**‒**9** | MD04G1226400 | probable WRKY Transcription Factor 74 [*Malus domestica*] | 0 | 100.00 |
| **G**‒**10** | MD15G1191400 | Transcription Factor bHLH68-like [*Malus domestica*] | 0 | 100.00 |
| **G**‒**11** | MD03G1017700 | Receptor-like Protein 2 [*Malus domestica*] | 0 | 100.00 |
| **G**‒**12** | MD03G1022000 | G-type lectin S-receptor-like serine/threonine-protein kinase LecRK3 [*Malus sylvestris*] | 0 | 99.88 |
| **G**‒**13** | MD12G1189900 | WRKY DNA-binding Transcription Factor 70-like [*Malus domestica*] | 1.0×10^-140^ | 99.48 |
| **G**‒**14** | MD05G1218000 | G-type lectin S-receptor-like Serine/threonine-protein Kinase At4g27290 isoform X1 [*Malus domestica*] | 0 | 95.48 |
| **G**‒**15** | MD16G1212600 | Ethylene Receptor 2 isoform X1 [*Malus domestica*] | 3.0×10^-125^ | 96.39 |
| **G**‒**16** | MD00G1007000 | probable LRR Receptor-like Serine/threonine-protein Kinase RFK1 isoform X1 [*Malus domestica*] | 0 | 89.20 |
| **G**‒**17** | MD10G1313800 | Cysteine-rich Receptor-like Protein Kinase 25 isoform X1 [*Malus domestica*] | 0 | 100.00 |
| **G**‒**18** | MD09G1035700 | LRR Receptor-like Serine/threonine-protein Kinase [*Pyrus ussuriensis* × *Pyrus communis*] | 0 | 84.02 |
| **G**‒**19** | MD10G1206800 | Cysteine-rich Receptor-like Protein Kinase 10 [*Malus domestica*] | 0 | 100.00 |
| **G**‒**20** | MD07G1255200 | Receptor Kinase-like Protein Xa21 [*Pyrus x bretschneideri*] | 1.0×10^-15^ | 90.48 |
| **G**‒**21** | MD02G1235600 | Leaf Rust 10 Disease-resistance Locus Receptor-like Protein Kinase-like 2.1 [*Malus domestica*] | 0 | 100.00 |
| **G**‒**22** | MD09G1190600 | IQ domain-containing Protein IQM1-like [*Malus domestica*] | 0 | 95.89 |
| **G**‒**23** | MD00G1125300 | probable Calcium-binding Protein CML45 [*Malus domestica*] | 2.0×10^-137^ | 100.00 |
| **G**‒**24** | MD06G1194700 | probable Calcium-binding Protein CML47 [*Malus domestica*] | 6.0×10^-150^ | 100.00 |
| **G**‒**25** | MD10G1185000 | Ethylene-responsive Transcription Factor ERF098 [*Malus domestica*] | 5.0×10^-119^ | 100.00 |
| **G**‒**26** | MD06G1120000 | Transcription Factor MYC3-like [*Malus domestica*] | 0 | 100.00 |
| **G**‒**27** | MD02G1273500 | Rust Resistance Kinase Lr10-like isoform X1 [*Malus sylvestris*] | 0 | 88.82 |
| **G**‒**28** | Novel_G000135 | Tropomyosin-like [*Malus domestica*] | 4.0×10^-55^ | 71.21 |
| **G**‒**29** | MD00G1082400 | Receptor-like Protein 3 [*Malus domestica*] | 0 | 100.00 |
| **G**‒**30** | MD10G1307500 | G-type lectin S-receptor-like Serine/threonine-protein Kinase At1g61490 [*Malus domestica*] | 0 | 100.00 |
| **G**‒**31** | MD06G1080900 | probable Copper-transporting ATPase HMA5 [*Malus sylvestris*] | 0 | 98.26 |
| **G**‒**32** | MD01G1037100 | Transcription Factor bHLH94-like [*Malus domestica*] | 0 | 100.00 |
| **G**‒**33** | MD05G1198800 | Ethylene-responsive Transcription Factor ERF098-like [*Malus domestica*] | 2.0×10^-112^ | 100.00 |
| **G**‒**34** | MD12G1046000 | Calcium-dependent Protein Kinase 20-like [*Malus domestica*] | 0 | 100.00 |
| **G**‒**35** | MD12G1253000 | probable Leucine-rich Repeat Receptor-like Protein Kinase At5g63930 [*Malus sylvestris*] | 0 | 87.06 |
| **G**‒**36** | MD15G1076500 | putative Receptor Protein Kinase ZmPK1 [*Malus domestica*] | 0 | 100.00 |
| **G**‒**37** | MD02G1181300 | probable esterase KAI2 [*Malus domestica*] | 0 | 100.00 |
| **G**‒**38** | MD14G1044700 | Calcium-dependent Protein Kinase 20-like [*Malus domestica*] | 0 | 100.00 |
| **G**‒**39** | MD10G1306600 | G-type lectin S-receptor-like serine/threonine-protein kinase B120 [*Malus domestica*] | 0 | 95.74 |
| **G**‒**40** | MD11G1262300 | Epidermis-specific Secreted Glycoprotein EP1-like [*Malus sylvestris*] | 0 | 99.32 |
| **G**‒**41** | MD12G1016000 | uncharacterized protein LOC103449381 [*Malus domestica*] | 3.0×10^-103^ | 100.00 |
| **G**‒**42** | MD06G1046300 | Serine/threonine-protein Kinase SAPK3-like [*Malus domestica*] | 0 | 100.00 |

| **Type of**  **TFs** | **Total number of TFs annotated** | **Number of TFs**  **with significant differences** | **Type of**  **TFs** | **Total number of TFs annotated** | **Number of TFs**  **with significant differences** |
| --- | --- | --- | --- | --- | --- |
| **MYB** | 243 | 9 | **PLATZ** | 15 | 2 |
| **bHLH** | 144 | 7 | **TCP** | 28 | 2 |
| **AP2**‒**EREBP** | 175 | 4 | **WRKY** | 106 | 2 |
| **NAC** | 132 | 4 | **C2C2**‒**GATA** | 71 | 1 |
| **ABI3VP1** | 63 | 3 | **GRAS** | 74 | 1 |
| **MADS** | 62 | 3 | **LOB** | 45 | 1 |
| **C2C2**‒**Dof** | 43 | 2 | **SBP** | 27 | 1 |
| **C2H2** | 71 | 2 | **TIG** | 6 | 1 |
| **GRF** | 14 | 2 | **Trihelix** | 52 | 1 |
| **OFP** | 18 | 2 | **zf**‒**HD** | 17 | 1 |

**Table S3 Number of upregulated and downregulated TFs induced by the overexpression of *MdKAI2***

**Table S4 Primers used in this study**

| **Primers used for gene cloning** | | |
| --- | --- | --- |
| **Gene ID** | **Forward primer** | **Reversed primer** |
| **MD02G1181300** | CGGGATCCATGTGGTGCCAGTTGGGACCT | GAAGGCCTTCAGATATGACATAGCCGTG |
| **Primers used for qRT‒PCR assays** | | |
| **Gene ID** | **Forward primer** | **Reversed primer** |
| **MD02G1181300** | CTAACCAACAATCAAGCAACAG | TGCTTCCAGACCGACTGATCG |
| **MD14G1044700** | ATGGTTTGGAAAGAGTGGGT | CACTGTTGTCCACATCCGC |
| **MD12G1046000** | TGAGTAGAAAGGTAGCGTGAGTAT | CCCCTGAAATCTGGCTGA |
| **MD04G1226400** | GCTTTGAAGACCCTGCTATG | GCCTAAGTTTGTTCCGATTG |
| **MD14G1127000** | ACTCCAGCCAAAGCACCA | ACATCCACTTCCACAGCCG |
| **MD12G1095300** | GACGCCTCACCTCAAGATAGA | AGGGAGGCTTTGTCTGTCTT |
| **MD13G1252700** | TACTGGTCCAACATTTTCGG | GGTGAGGAATTGTTACCATAAGAC |
| **MD02G1190000** | AATGTTTCGCAAATCGGC | TCTGAGTTTCGGTGCTTGG |
| **MD10G1185000** | AAGAACCGAAGGCACGAG | ATAAGCCCTTGCTGCCTC |
| **MD05G1198800** | AATAACCAGCAGAGGGAGCC | CGTGCTCCATTTCTCGTAGG |
| **MD16G1277000** | GCAAGGTTGTTTGTCAATCTG | TCATCGGTGAGGCGGTA |
| **MD14G1110800** | ACAACGAGATCAAGAACTACTGG | TAGGTGGTGGACTTATTGTGG |
| **MD15G1051400** | CGGGGCGAACAGACAAT | GGATTTGCTTGGATGTTGAGA |
| **MD06G1192900** | TGTCTCGGAGAATTGCCAA | CATCTCAGCAAACCAGTGTTAG |
| **MD04G1184900** | TCCTCATCACAATAACCAAAGC | TCGGATGGGAGGCTGAT |
| **MD01G1094000** | GGGAACACTGGGAAGCAA | GAGCAGCCAAATCCCAAG |
| **MD01G1037100** | AATAAGGTAGGGCAACAAGGT | GGCAGCAAGATAGACATTCATT |
| **MD12G1189900** | CATTCGCTACCCAGTGATTATC | AAAGACAGACTCGGCAAACA |
| **MD11G1262300** | GCTTTCACCCACATTTACCA | CTGAGCGAGTCGTACCCAT |
| **MD05G1294500** | TCGGGGCGTGTCAATT | GCTTAGCCAAACCAAAGTCAG |
| **MD03G1242600** | TGAGTAGAAAGGTAGCGTGAGTAT | CCCCTGAAATCTGGCTGA |
